# Present and future projections of habitat suitability of the Asian tiger mosquito, a vector of viral pathogens, from global climate simulation

**DOI:** 10.1098/rstb.2013.0554

**Published:** 2015-04-05

**Authors:** Y. Proestos, G. K. Christophides, K. Ergüler, M. Tanarhte, J. Waldock, J. Lelieveld

**Affiliations:** 1 Computation-based Science and Technology Research Center (CaSToRC), The Cyprus Institute, 20 Konstantinou Kavafi Street, 2121 Aglantzia, Nicosia, Cyprus; 2 Faculty of Natural Sciences, Department of Life Sciences, South Kensington Campus, Imperial College London, London SW7 2AZ, UK; 3 Department of Atmospheric Chemistry, Max Planck Institute for Chemistry, Hahn-Meitnerweg 1, 55128 Mainz, Germany

**Keywords:** *Aedes albopictus*, global climate modelling, habitat suitability, climate change, vector distribution model, vector-borne diseases

## Abstract

Climate change can influence the transmission of vector-borne diseases (VBDs) through altering the habitat suitability of insect vectors. Here we present global climate model simulations and evaluate the associated uncertainties in view of the main meteorological factors that may affect the distribution of the Asian tiger mosquito (*Aedes albopictus*), which can transmit pathogens that cause chikungunya, dengue fever, yellow fever and various encephalitides. Using a general circulation model at 50 km horizontal resolution to simulate mosquito survival variables including temperature, precipitation and relative humidity, we present both global and regional projections of the habitat suitability up to the middle of the twenty-first century. The model resolution of 50 km allows evaluation against previous projections for Europe and provides a basis for comparative analyses with other regions. Model uncertainties and performance are addressed in light of the recent CMIP5 ensemble climate model simulations for the RCP8.5 concentration pathway and using meteorological re-analysis data (ERA-Interim/ECMWF) for the recent past. Uncertainty ranges associated with the thresholds of meteorological variables that may affect the distribution of *Ae. albopictus* are diagnosed using fuzzy-logic methodology, notably to assess the influence of selected meteorological criteria and combinations of criteria that influence mosquito habitat suitability. From the climate projections for 2050, and adopting a habitat suitability index larger than 70%, we estimate that approximately 2.4 billion individuals in a land area of nearly 20 million km^2^ will potentially be exposed to *Ae. albopictus*. The synthesis of fuzzy-logic based on mosquito biology and climate change analysis provides new insights into the regional and global spreading of VBDs to support disease control and policy making.

## 1. Introduction

Public health is very likely to be adversely impacted by climate change [1]. Future climate projections, even under optimistic emission scenarios, indicate a substantial rise in near-surface temperatures and changes in the hydrological cycle. As a result, the incidence frequency of vector-borne diseases (VBDs) may be affected (increase or decrease) through differences in the geographical distribution and breeding seasons of the insect vectors that transmit them.

In the past three decades *Ae. albopictus* (Skuse, 1894), an invasive and easily adapting species (it may reproduce in flower pots, tin cans, bird baths, used tyres, tree-holes, etc.), has rapidly dispersed from its native Southeast Asian occupancy by establishing populations in parts of Europe, Africa, North and South America [2]. *Ae. albopictus* is a proven vector of dengue (DENV), chikungunya (CHIKV) as well as West Nile arboviruses (under field conditions) [3–5]. Under laboratory conditions *Ae. albopictus* is capable of transmitting more than 20 viruses, dangerous to both human and (domesticated) animal populations [5,6]. Therefore, the fast pace of its geographical spreading, its high ecological and physiological adaptability and its potential efficacy of transmitting hazardous pathogens makes *Ae. albopictus* a serious public health threat, especially for vulnerable populations [7,8].

Considering its potential threat to public health, several studies have been conducted to estimate the possible spatial distribution of *Ae. albopictus* using species distribution models driven by environmental variables. For instance, the European Center for Disease Prevention and Control (ECDC) studied climate change impacts using different IPCC emission scenarios [9] and derived risk maps of the possible future habitat distribution of *Ae. albopictus* over the European continent [10], using among others, a multi-criteria decision model driven by meteorological variables. Also focusing on Europe, Caminade *et al.* [11] compared the results from three different vector distribution models driven by meteorological variables computed by regional models and assessed the spatial distribution of the habitat suitability of *Ae. albopictus* under recent and future climate conditions. Another study on the geographical spread of *Ae. albopictus* over Europe under different climate conditions by Fischer *et al.* [12] employed machine learning and expert knowledge to model the vector distribution. Rochlin *et al.* [13] investigated how climate change is expected to impact the habitat of *Ae. albopictus* in selected areas of the northeastern United States. Moreover, a recent study by Fischer *et al.* [14] summarized and compared mechanistic and statistical/correlative niche approaches employed to predict the geographical distribution of the vector, mainly considering the European continent. This study indicated that investigation of spatial characteristics (e.g. introduction gateways, dispersal pathways) and further laboratory experiments assessing the meteorological constraints/thresholds affecting the mosquito are necessary to improve modelling results. These studies consistently point to a redistribution in the habitat range of the mosquito as a consequence of the projected warmer and wetter winters, and warmer and drier summers in the geographical regions considered.

It is important to mention that besides studies on the influence of climate change on the geographical expansion of the vector, research has been carried out regarding the risks of epidemic outbreaks related to chikungunya and dengue fever, viral infections transmitted by the Asian tiger and *Aedes aegypti* mosquitoes. In early work, Poletti *et al.* [15] developed a climate-driven model of CHIKV transmission. Tilston *et al.* [16] combined climatological information and data of the chikungunya outbreak that occurred in Italy during the summer of 2007 to identify risk surveillance zones at a European level. A similar study by Fischer *et al.* [17] using two climate change scenarios and previous results for the habitat suitability of *Ae. albopictus* over Europe, chikungunya, projecting that France, northern Italy and East-Central Europe have the highest risk of CHIKV transmission by the end of the twenty-first century. The global distribution and burden under dengue fever has recently been re-examined by Bhatt *et al.* [18], producing updated risk maps for the transmission of the disease, estimating that there are approximately 390 million dengue cases per year, a number that is more than three times higher than the current estimate by the World Health Organization. Finally, Delmelle *et al.* [19,20] developed tools based on local weather, climate and epidemiological data to forecast seasonal dengue fever outbreaks in the urban environment of Cali, Colombia. To date, few data are available to inform epidemiological models of CHIKV transmission, although more data are present in the literature on DENV transmission, making accurate modelling of disease transmission extremely difficult. This is an area requiring further experimental work in the laboratory and the field.

In this study, we hypothesize that future climate change scenarios will influence the global distribution of *Ae. albopictus* through changes in global temperatures and shifting in precipitation patterns. Projecting the spatio-temporal distribution of disease vectors under climate changing conditions requires reliable modelling of vector populations and robust climate models that produce the corresponding meteorological variables in accord with the present climate conditions as well as being compliant with future projections. In recent years, climate modelling has achieved significant progress and the computational resources have increased. However, sufficiently high-resolution simulations on a global scale remain limited, mainly owing to the vast computational resources required.

Here we present, for the first time, fairly high resolution globally consistent habitat suitability maps of the Asian tiger mosquito for present and projected future climate conditions. These maps were created using a vector distribution model driven by seven meteorological criteria following a (fuzzy-logic) multi-criteria decision analysis method. To derive the seven meteorological variables, we have performed global climate simulations at a relatively high spatial resolution of approximately 50 km (at the Equator) by employing the EMAC general circulation model (GCM) [21–23]. Although finer resolution regional studies, for instance at 25 km [11] covering Europe, and studies modelling the *Ae. albopictus* distribution locally over northeastern Italy and Switzerland [24,25] have been performed, the spatial resolution is close to that of regional studies, notably over Europe, and thus the results can be directly compared. The climate simulations have been carried out for two time periods, one that represents the recent past (reference period) spanning years between 2000 and 2009, and the future projections spanning years between 2045 and 2054. Enhanced resolution global simulations typically improve the climate projections because of the more realistic description of the topography, such as mountainous and coastal regions, and associated atmospheric flows [12]. Since this is the first global assessment of its kind, we devote particular attention to evaluating climate model uncertainties from the perspective of the so-called robustness metric, introduced recently by Knutti *et al.* [26], also employing the Intergovernmental Panel on Climate Change (IPCC) CMIP5 multi-model ensemble. Furthermore, we present a direct comparison between the climate data extracted from our EMAC model simulations, and the ERA-Interim (ECMWF) re-analysis meteorological dataset for the recent period [27].

## 2. Models and methodology

### (a) Climate model

Temperature, precipitation and relative humidity (RH) are among the important meteorological variables that affect the ecology and habitat suitability of the Asian tiger mosquito [5]. To compute the relevant variables we have employed the ECHAM5/MESSy2 atmospheric chemistry (EMAC) GCM using a high-resolution mode. EMAC includes sub-models describing tropospheric and middle atmospheric processes and their interactions with oceans, land and vegetation, and trace species emissions of natural and anthropogenic origin [21–23,28].

For the present global climate simulations we utilized a horizontal grid spacing (T255) that resolves 768 longitude times 384 latitude points, where grid boxes, at the Equator, have a horizontal dimension of approximately 50 × 50 km^2^—corresponding to a quadratic Gaussian grid of approximately 0.47° × 0.47° in latitude and longitude—and 31 layers in the vertical dimension that represent pressure levels up to 10 hPa (lower stratosphere).

Two 10-year simulation runs have been performed for the reference period (2000–2009) and the future projection period (2045–2054). For the former simulation, we have imposed as climatic boundary conditions the AMIP-II [29] sea-surface temperature (sst) and sea-ice coverage (sic) assimilation data, while the simulation for the future projections uses sst and sic boundary conditions derived from the IPCC SRES-A2 emissions scenario [9,30]. Briefly, the SRES-A2 scenario assumes intermediate to high future CO_2_ emissions and describes a hypothetical politically fragmented world where nations are self-reliant with economic development occurring on a regional scale and in which the world population is continuously growing. The EMAC climate model was set up with a 5 hour temporal resolution data output, chosen for optimal data sampling and representation of the diel cycle.

The performance of the EMAC model for the purposes of this study was tested against the European Centre for Medium-range Weather Forecasts (ECMWF) ERA-Interim (EI) re-analysis data [27,31]. Briefly, the EI data rely on globally recorded meteorological observations that are further processed through a model assimilation system, which uses a computationally demanding four-dimensional variational algorithm [32] that produces short range forecasts in order to determine the most realistic atmospheric state of the Earth.

### (b) CMIP5 multi-model ensemble and robustness

Producing multi-year global climate simulations from ensemble runs on a fine spatial resolution requires immense computational means in terms of CPU and financial investment, which is currently not permitted by the resources available to us. Therefore, in addition to the high-resolution data described in §2a, we have also considered the publicly available lower resolution global simulations from the Coupled Model Intercomparison Project–Phase 5 (CMIP5) [33]. The CMIP5 multi-model ensemble was selected to address model uncertainties based on the recently introduced metric of robustness [26]. A list of the CMIP5 models included in this study is shown in table 1.

**Table 1.** CMIP5 models and the corresponding groups considered for the evaluation of the robustness measure *R*. Owing to inconsistent grid sizes, we have interpolated (regridded) the data to a common resolution of approximately 200 km (i.e. 1.875° × 1.86° in longitude and latitude). The following daily variables have been used: mean near-surface air temperature (tas), maximum near-surface air temperature (tasmax), minimum near-surface air temperature (tasmin) and precipitation (pr). The data have been downloaded from the ESGF database [34].

modelling centre (or group)	institute ID	model name	historical ensemble members	RCP8.5 ensemble members	longitude grid size (°)	latitude grid size (°)
National Center for Atmospheric Research	NCAR	CCSM4	3	3	1.25	0.94
Met Office Hadley Centre	MOHC	HadGEM2-CC, HadGEM2-ES	3	3	1.875	1.25
Institut Pierre Simon Laplace	IPSL	IPSL-CM5A-MR	3	1	2.5	1.27
Atmosphere and Ocean Research Institute (University of Tokyo), National Institute for Environmental Studies, and Japan Agency for Marine-Earth Science and Technology	MIROC	MIROC5	3	3	1.41	1.41
Max Planck Institute for Meteorology	MPI-M	MPI-ESM-LR	3	3	1.875	1.86
Meteorological Research Institute	MRI	MRI-CGCM3	3	1	1.125	1.125
Commonwealth Scientific and Industrial Research Organization in collaboration with Queensland Climate Change Centre of Excellence	CSIRO-QCCCE	CSIRO-Mk3.6.0	5	3	1.875	1.86
EC-EARTH consortium	EC-EARTH	EC-EARTH	3	3	1.125	1.125

It should be mentioned that when our global high-resolution climate simulations were carried out, the CMIP5 multi-model simulation results and the corresponding forcings of greenhouse gas concentrations and emission pathways, known as representative concentration pathways (RCP), were not yet available. Thus, for our model simulations regarding the future climate projections we applied the boundary conditions from the SRES-A2 emissions scenario. When the CMIP5 dataset became available, we selected the results that follow the RCP8.5 pathway, which corresponds to a radiative forcing of 8.5 W m^−2^ in 2100, and according to Riahi *et al.* [35,36] represents an extension and improvement of the SRES-A2 emissions scenario. Moreover, the RCP8.5 pathway serves as an upper bound of the RCPs; hence it is considered to be a pessimistic scenario. We have used the corresponding daily datasets as a basis for analysing the CMIP5 datasets.

The study of model uncertainties is performed by means of a recently introduced metric in climate studies, the so-called robustness factor *R* [26,37]. This metric, which is applied extensively in weather forecasting ensemble validation [38], provides a direct way of evaluating locally inter-model agreement on computed climate variables. For instance, using the selected models shown in table 1 and also including our EMAC results, we test the (dis)agreement of calculated near-surface temperature and precipitation, both key driving variables for the mosquito spatial distribution model.

### (c) Fuzzy-logic and habitat constraints

Based on and extending previous work [3,5,39–42] on the environmental/climatic factors affecting the life cycle of the Asian tiger mosquito, a multi-criteria decision analysis vector distribution model has been devised to estimate the global habitat suitability maps. The mosquito spatial distribution model combines seven meteorological indices based on field observations, extensive literature review [5] and expert knowledge. The model serves as a tool to explore and identify the geographical areas that can potentially sustain the thriving of the mosquito in recent and future periods, i.e. under climate change scenarios.

As discussed at length in Waldock *et al.* [5], selecting thresholds of environmental criteria for defining species distributions should take into account the scale and accuracy of the climate model used. While laboratory experiments may indicate the upper and lower limits of *Ae. albopictus* survival and development, these conditions will not be found uniformly across one grid square in any model output. Even with relatively high-resolution model output of 50 km, micro-climates will exist within each grid square. The thresholds for our environmental criteria have been selected based on comparison of EMAC model outputs with existing populations of *Ae. albopictus* in Europe as described in Waldock *et al.* [5]. The seven empirical criteria used to define a suitable, in terms of climate conditions, environment for the Asian tiger mosquito using EMAC model outputs are

(a) The annual average precipitation is at least 200 mm. Precipitation is a complex parameter to model for *Ae. albopictus* populations as breeding sites for this species can be independent of rainfall. Despite a threshold of 500 mm being reported previously [43], *Ae. albopictus* populations are confirmed in areas of Spain where annual rainfall is 292 mm [44]. Using existing European *Ae. albopictus* population distribution, as in Waldock *et al.* [5], we selected a mean annual cut-off of 200 mm for our analysis.
(b) The annual average temperature is higher than 8.0°C. Laboratory work analysing the temperature dependence of *Ae. albopictus* survival and development [45–47] gives lower thresholds for development of 12.8°C, 12.5°C and 9.6°C for eggs, larvae and pupae, respectively [5]. Survival thresholds are around 10–40°C and 10–37°C for larvae and pupae, respectively [5]. To simplify our analysis, we use the average annual temperature to define area suitable for mosquito survival and development. Using existing European *Ae. albopictus* populations as in [5], we selected an annual temperature cut-off of 8.0°C.
(c) In January of the Northern Hemisphere (NH) (July of the Southern Hemisphere (SH)) minimum temperature is above −4.0°C. Minimum temperatures for *Ae. albopictus* egg survival are usually given as average January temperatures. In the literature, lower limits for *Ae. albopictus* range from −3.0°C to −5.0°C in China [48,49], 0°C to −2.0°C in Japan [49], 0°C to −5.0°C and −2.0°C in North America [13,48]. As for the previous criteria, using European populations of *Ae. albopicus* as in Waldock *et al.* [5], we selected a cut-off of −4.0°C.
(d) The summer maximum temperature does not exceed 40.0°C. Studies show that at 40°C eggs fail to hatch [7], and fitting curves to survival data from multiple studies (as carried out in Waldock *et al.* [5]) gives upper limits of survival around 40.0°C.
(e) At least 60 days have measurable (greater than 1 mm) rainfall. A minimum of 60 days with precipitation per year has been used by Roiz *et al.* [42], Erijta *et al.* [44], Benedict *et al.* [3], and this is in agreement with EMAC model outputs compared with European *Ae. albopictus* population as depicted in [5].
(f,g) The summer RH is at least 30% and the winter RH is 50% or higher. RH primarily affects the adult and egg stages of *Ae. albopictus*. Limited data in the literature demonstrate that survival and egg hatch rates are improved at higher RH [50,51]. Between 60% and 90%, little difference is observed in adult survival [39,46,47,52,53]. We have selected our thresholds using EMAC model outputs and European *Ae. albopictus* distribution as in Waldock *et al.* [5]. Populations are established in regions with summer RH as low as 35%, demonstrating the wide variety of RH tolerated by the species.

The global maps presenting each one of the predictor variables (obtained from the EMAC simulation data) are given in the electronic supplementary material, figures S1 and S2 (recent past), and figures S3 and S4 (future projection).

**Figure 1.**
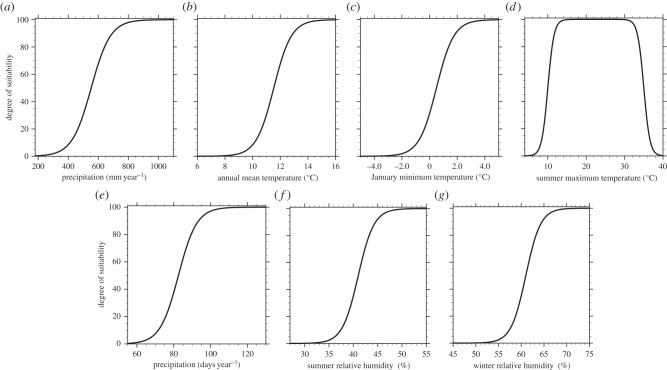
Sigmoidal membership functions defining the degree of suitability: (*a*) annual mean precipitation, (*b*) annual mean temperature, (*c*) January-NH (July-SH) minimum temperature. (*d*) For the summer, June–August for NH (December–February for SH) maximum temperature, a symmetric sigmoidal function (difference between two sigmoidal functions) has been used so that the optimal suitability is approximately between 16 and 32°C, and the survival chance is zero for temperatures above 40°C. (*e*) Days of precipitation per year, (*f*) summer, i.e. June–August for NH (December–February for SH) RH, and (*g*) winter, i.e. December–February for NH (June–August for SH) RH. The associated maps representing the suitability scores for each of the standardized meteorological variables are provided in the electronic supplementary material, figures S5 and S6, and figures S7 and S8, for both recent past and future periods, respectively.

As the multi-criteria model (effectively) introduces sharp thresholds on the considered climatic variables, which is considered unrealistic, we follow fuzzy-logic based on sigmoidal membership functions. The latter are continuous (smooth) functions that can be used to express the degree of suitability of a certain meteorological variable around a threshold. Hence, any variability associated to the various thresholds, imposed on the variables affecting the habitat suitability of the vector mosquito, is fixed upon the introduction of the sigmoidal membership functions. All the habitat/predictor variables are standardized according to a sigmoidal function of the form2.1
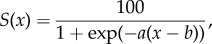
except for the summer maximum temperature variable, which uses a symmetric combination of sigmoidal functions. *S*(*x*) expresses the degree of suitability, where *x* is the predictor variable under consideration (e.g. annual mean precipitation), while *a*, *b* are characteristic constants (determining the rate of change of the function as it passes through its mid-point and the location of its point of inflection, respectively), whose values are chosen accordingly to conform with the associated constraint. The upper-bound score of the sigmoidal functions is chosen to be 100. Our process-based (mechanistic) approach is analogous to that followed by the ECDC [10] and also explored in Caminade *et al.* [11], who studied the distribution of *Ae. albopictus* over Europe, using observations and results from regional climate simulations. Our multi-criteria decision model for the vector spatial distribution, apart from using global climate simulation data for the vector habitat suitability, also introduces more constraints in the sense that it includes seven climatic factors (including extreme winter and summer temperatures) that are combined to produce the habitat suitability maps. The fuzzy-logic based multi-criteria decision model for projecting the vector geographical dispersal is illustrated by the set of sigmoidal function plots provided in figure 1.

By applying these constraints to the global climate data derived from our EMAC simulation, we estimate the geographical areas that could potentially sustain the establishment/colonization of mosquitoes (assuming they are introduced) during the reference period (years 2000–2009), and further help us identify the regions that can support the presence of the mosquito in the projected future period (years 2045–2054) under the SRES-A2 emissions scenario. After rescaling each of the seven habitat variables using the sigmoidal functions, we define the habitat suitability index^1^ (hsi), which is a measure of the possible spatial (habitat) distribution of the Asian tiger mosquito. In this study, the habitat suitability score, *S*, is calculated using an equal weight, geometric mean combination of the seven (predictor variables') suitability scores *S_i_*2.2
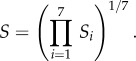


Besides tuning the sigmoidal membership function parameters, one could elaborate and refine the weights chosen for the seven criteria by applying a fuzzy analytic hierarchy process (AHP) [54–56], where a panel of experts on the field classifies the several criteria according to their significance.

The urban heat island effect [57–59] was taken into account in the estimation of the habitat suitability index considering that *Ae. albopictus* thrives in cities. The effect was considered *a posteriori*, as the EMAC climate model does not have a mechanism for simulating the phenomenon. Using (gridded) population data [60,61], centred at the years 2005 and 2050 for the two simulation periods considered, we increased the temperature, on average, by 1°C in (urban) areas with a population density equal to or greater than 400 individuals km^−2^, the latter leading to a global population fraction of 50% in 2005.

## 3. Results

First, we briefly present a comparison of the near-surface temperature between the EMAC (T255) simulations for the reference period and the corresponding EI dataset. Subsequently, we present the results regarding the mosquito habitat suitability driven by the multi-criteria decision model from using the two high-resolution simulation datasets. Based on the CMIP5 multi-model ensemble, we then discuss uncertainties in climate model projections, focusing on robustness—a measure that examines the multi-model ensemble agreement—using two fundamental climatic factors affecting the mosquito habitat, i.e. the near-surface temperature and precipitation in the part of the globe that is relevant for *Ae. albopictus* (i.e. excluding oceans and land areas with less than 10% hsi). Furthermore, we discuss the hsi in view of the recently produced CMIP5 multi-model ensemble database.

### (a) EMAC compared to ERA-Interim

**Figure 2.**
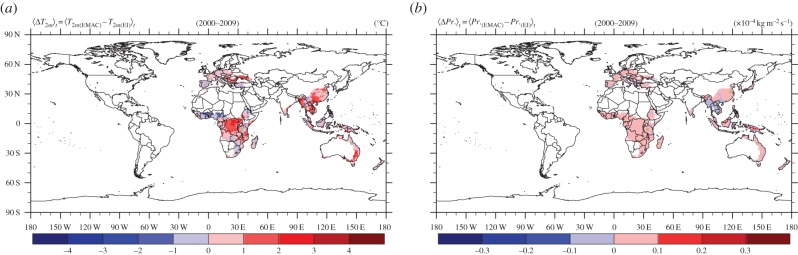
Comparing the ERA-Interim (EI) data with the model simulation over the period 2000–2009. (*a*) Mean near-surface temperature difference. (*b*) Average precipitation flux difference. Areas with hsi less than 10% are not considered. The same maps but with all areas included are shown in the electronic supplementary material, figures S12 and S13.

**Figure 3.**
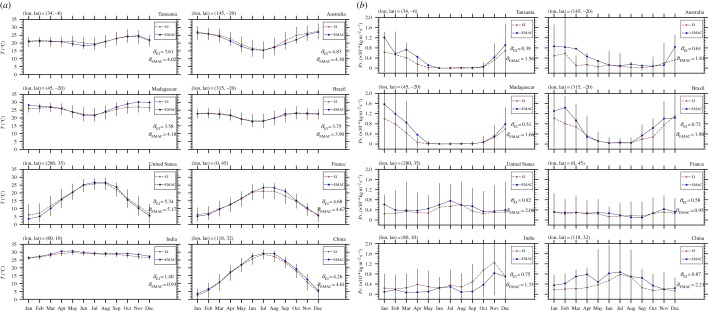
Monthly climatologies from the ERA-Interim (EI) and the model simulation data over the 2000–2009 period, for selected locations where *Ae. albopictus* presence is expected. (*a*) Near-surface temperature and (*b*) precipitation flux. The vertical axes are on the same scales for better comparison and error bars on the EI values represent two standard deviations. The selected locations are shown with green dots in figure 2.

The scarcity of global gridded observational data for the meteorological fields, required for the vector distribution model, makes the evaluation of our high-resolution global datasets rather difficult. However, the EI dataset is considered the best alternative for comparison. The EI dataset is provided on a coarser spatial grid than our climate model, corresponding to a horizontal resolution of approximately 75 km (at the Equator), where data were stored with a frequency of 6 hours. First, we evaluate our results against the near-surface temperature field from the EI dataset for the relevant period of 2000–2009. This was done after our data had been (bi-linearly) interpolated in time and in grid space, respectively. Figure 2*a* displays the mean difference over the 10-year period (2000–2009). For the two results, we find a highly significant spatial (grid space) correlation, with Pearson's *r*-test correlation coefficient^2^, *r* = 0.99 [99.9%CI, *p* < 0.001] showing that differences are generally small. Monthly climatologies for relevant locations (green dots in figure 2) in all continents, for the decade 2000–2009, are shown in figure 3*a*. The comparison of the EMAC computed and EI assimilated seasonal cycles corroborates the good agreement, well within the 1*σ* variability, although in individual locations small biases can occur, which are, however, unlikely to have a significant effect on the results of the habitat suitability calculations.

Similarly, we performed a comparison for the precipitation flux. The two datasets are relatively strongly correlated with *r* = 0.87 [99.9% CI, *p* < 0.001]. Figure 2*b* depicts the mean difference over the 10-year period (2000–2009), indicating relatively small discrepancies. The comparison for individual locations in figure 3*b* shows that the rainfall seasonalities typically compare well, although biases can be much larger than for temperature. As precipitation fluxes are highly variable (figure 3*b*) and because uncertainties are larger than for temperature, it may be desirable to test the sensitivity of the vector distribution model to uncertainties associated with individual predictor variables in future.

For completeness, we have included graphs comparing EMAC against EI data, along with regression lines for both the near-surface temperature and precipitation fields in the electronic supplementary material, figure S14.

### (b) Habitat suitability maps

Extending previous studies of the habitat suitability of *Ae. albopictus* with a regional focus, here we employ the global GCM EMAC to simulate climatic factors that influence *Ae. albopictus* spatial distribution. The results from the relatively high-resolution global analysis can be compared with previous work for Europe and are additionally relevant for other parts of the world.

**Figure 4.**
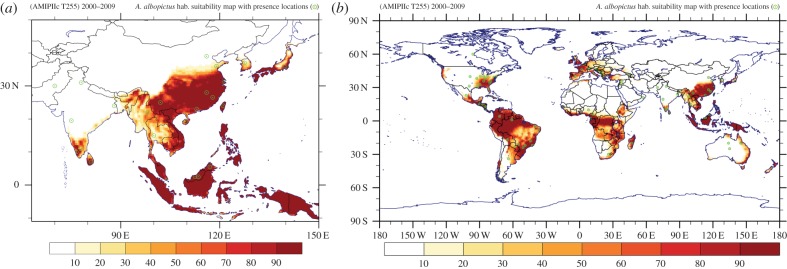
(*a*) Southeast Asian and (*b*) global maps of the habitat suitability based on the high-resolution climate model results (T255) for the recent period 2000–2009. The (green) dots show the locations where presence of *Ae. albopictus* has been reported according to the CABI database.

**Figure 5.**
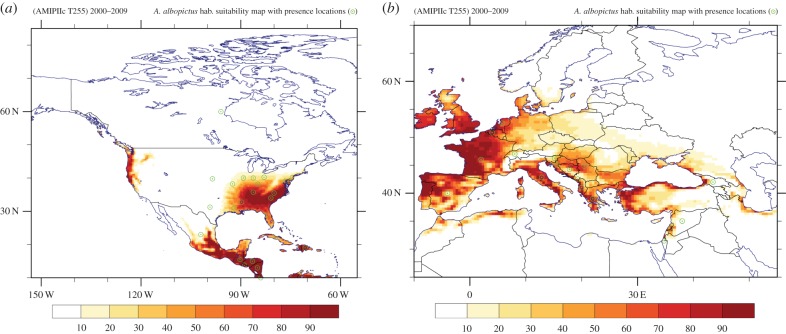
(*a*) North American and (*b*) European maps of the habitat suitability based on the high-resolution climate model results (T255) for the recent period 2000–2009. The (green) dots show the locations where presence of *Ae. albopictus* has been reported according to the CABI database.

Figure 4*a* shows that the *Ae. albopictus* climatically suitable region of Southeast Asia is captured by our vector distribution model, with an hsi above 90% in most areas. Owing to the lack of publicly available geospatial (gridded) data of the current presence or absence of *Ae. albopictus* outside its native range of Southeast Asia, the assessment of our vector distribution model results is limited. However, a qualitative evaluation can be conducted based on online information made available by CABI, a non-profit international organization concerned with environmental issues [2,62]. Following a simple analysis approach, by comparing the global occurrence (e.g. location coordinates) [2] against the corresponding ones from the habitat suitability map, it is evident that approximately 70% of the (sparse) geo-referenced data from the CABI database occur in the region of at least 35% hsi, estimated by the species' spatial distribution model. The several misses in non-climatically suitable locations (e.g. western India) are possibly associated with the fact that urban environments, which may help reproduce the necessary climatic conditions for mosquito establishment (e.g. providing water containers in dry areas), have not been explicitly considered in our vector distribution model. The coincidence with the vector distribution model prediction, under the current climatic conditions, is depicted in figures 4 and 5. Apart from the fact that a number of points reported in the CABI database refer only to the country level, it should also be noted that in some of the reported locations *Ae. albopictus* may have already been eradicated or is under control. Therefore, it will be essential for the community to establish a global network that reliably monitors the vector distribution, and the data should be publicly available.

The global map in figure 4*b* shows that central and southern Africa (including the tropical rainforest) and equatorial coastal western Africa, approximately between latitudes 8° N and 16° S, currently provides suitable conditions for establishment of the vector. This is corroborated by recent disease outbreaks related to *Ae. albopictus* reported in Nigeria [2,5]. Suitable habitat conditions for the mosquito also seem to be provided by the coastal areas of southeastern Africa. A highly suitable environment is also found in tropical southcentral America, especially along the eastern coastal part of Brazil. Within the United States, depicted in figure 5*a*, the model identifies most of southeastern states as suitable habitat areas for the mosquito. Furthermore, we find quite suitable conditions for vector survival in the populated southeast coastal area of Australia and the North Island of New Zealand. This provides support for the strict measures that have been implemented in many developed countries with a currently high degree of habitat suitability to prevent the establishment and distribution of *Ae. albopictus*. Such measures are also needed in less developed countries with high habitat suitability.

Figure 5*b* depicts the recent habitat suitability in the European and Mediterranean basin area, indicating that regions such as the central part of Italy, most of Greece, Albania, Montenegro, Croatia, Bosnia and Herzegovina, western and northern coastal Turkey, have highly suitable climatic conditions for the establishment of *Ae. albopictus* were it to be introduced. It is also quite notable that the part of Israel where the mosquito has been reported is climatically suited as calculated with the high-resolution model. In §3(d), it will be shown that such locations cannot be resolved at a coarse grid scale. Figure 5*b* also shows that parts of the Iberian peninsula, France, southern England and Ireland could actually be hot spots for a potential establishment as the current habitat conditions appear to be highly suitable for the vector, although it has not been observed or reported (yet). The low degree of suitability encountered in countries such as central-eastern Germany, Czech Republic, Slovakia, Poland and extending to Ukraine is attributed mainly to the winter minimum temperature and the relatively low annual average temperatures in these areas. It should be mentioned that sometimes *Ae. albopictus* has been observed in cities where our model predicts less suitable conditions. This is probably related to very localized urban environs where temperature and precipitation thresholds are being circumvented, such as in parks, gardens, etc. It might be necessary to define adjusted mosquito survival criteria for such conditions to be addressed in future work. From figure 5*b*, it is also notable that in the Mediterranean region coastal areas appear to be suitable habitats, related to the cooling influence of the sea in summer, which prevents temperatures exceeding 40°C in summer (also around the Caspian and Black Seas). On the other hand, in elevated areas, e.g. in the Balkans and Anatolia, winters can be cold which precludes mosquito survival. Similarly, in distinct areas in other continents the habitat suitability of *Ae. albopictus* can be closely related to topographical features and coastlines, which underscores the usefulness of relatively high-resolution climate simulations.

**Figure 6.**
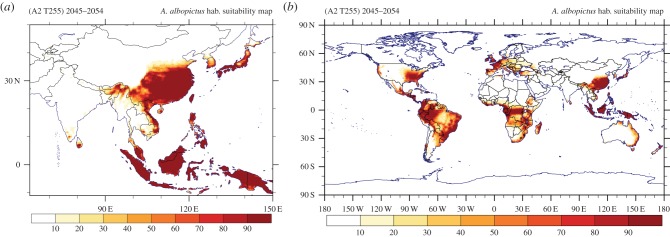
(*a*) Southeast Asian and (*b*) global maps of the simulated habitat suitability based on the future climate projections (SRES-A2 T255) over the period 2045–2054.

**Figure 7.**
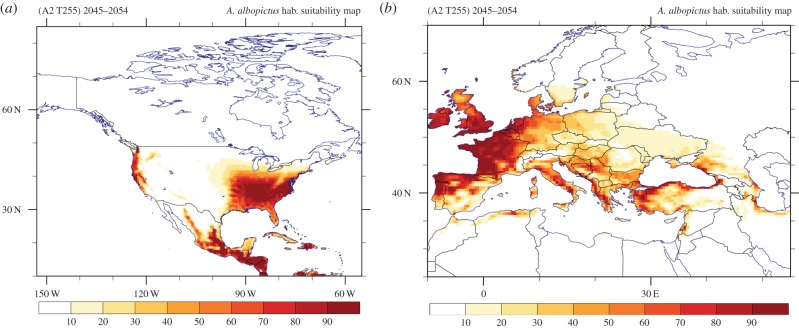
(*a*) North American and (*b*) European maps of the simulated habitat suitability based on the future climate projections (SRES-A2 T255) over the period 2045–2054.

Figures 6 and 7 show the hsi for the mid-century projection under the SRES-A2 emissions scenario. Thus, owing to the expected overall global warming, the changes in global climatic suitability are reflected in the shift/alteration of potentially suitable areas for invasion by and/or establishment of the *Ae. albopictus* vector. The projected climate change leads to significant pattern shifts in South America and in some parts of Southeastern Asia. Moreover, parts of the northern-central territories of Mexico are becoming more prone to mosquito establishment, whereas in the United States, the habitable area, apart from being more suited, expands further beyond the southern states to include mid-western states. Also notable is some expansion of the climatically favourable range in the western part of the United States. From figure 7*b*, an overall decrease in future habitat suitability is evident in southern European and Mediterranean areas, although the overall pattern seems mostly the same as in the corresponding reference period, while at the same time an increase in habitat suitability is projected in some of the northern and eastern European states.

In addition, habitat suitability plots for specific regions around the globe referring to both simulation periods are given in the electronic supplementary material, figures S9 and S10. Also maps obtained using the corresponding EI data showing the climatological fields, predictor variable suitability scores and differences in habitat suitability when compared against the EMAC-based estimate, have been included in the electronic supplementary material, figures S15 and S16, figures S17 and S18, and figure S19, respectively.

**Figure 8.**
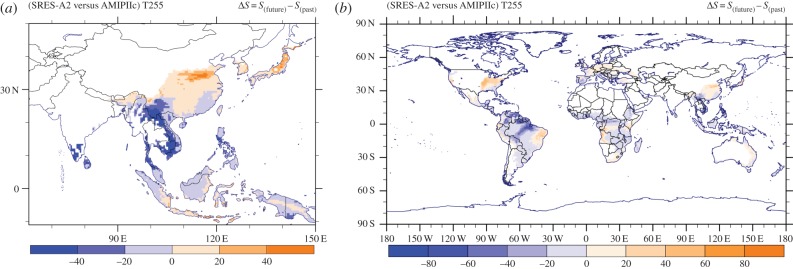
(*a*) Southeast Asian and (*b*) global maps of the habitat suitability change between the future and reference periods. Areas with hsi less than 10% have not been considered.

**Figure 9.**
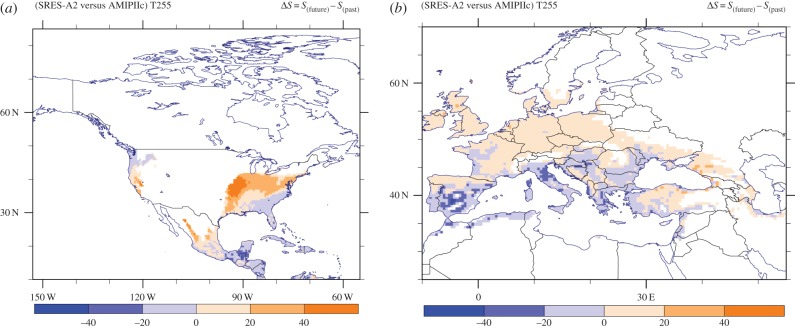
(*a*) North American and (*b*) European maps of the habitat suitability change between the future and reference periods. Areas with hsi less than 10% have not been considered.

The above results are summarized by the actual difference Δ*S* in habitat suitability pattern presented in figures 8 and 9. Note that before taking the difference between the fields, we excluded areas with hsi less than 10% as they are insignificant for mosquito survival. A pronounced decrease in habitat suitability is manifest in the South American tropical rainforest and savannah regions, representing a change in the suitable range by more than 50%. A similar decrease occurs in the southeastern Asian nations Laos, Vietnam and Cambodia, all in the *Ae. albopictus* native habitat zone. In contrast, we project an increased suitability over the northeastern United States, driven by the simulated warmer temperatures in future in conjunction with the increased RH. Overall, it appears that climate change induces a poleward shift of the suitable habitat conditions that is most apparent in Europe, the United States and eastern Asia, and to a lesser degree in southern Africa and Australia. For habitat suitability changes in specific regions around the globe, we refer to the electronic supplementary material, figure S11.

One conclusion can be readily drawn regarding the present and future climate scenarios. From the global habitat suitability maps for both the recent past and future projection, the regions with the most favourable conditions for mosquito establishment are, as expected, the tropical rainforest areas. This coincides in particular with the *Ae. albopictus* (original) native environment of Southeast Asia. Climate change seems generally to reduce habitat suitability in the tropical forest regions where *Ae. albopictus* is native. Owing to its high ecological plasticity, the decline of its native environment is unlikely to minimize its capacity to establish populations in other regions, especially in non-forested areas with dense human populations. Thus in response to climate change, where other regions become more suitable (than existing ones), mosquito populations may become established in these new territories. In future work, it may be desirable also to account for tropical deforestation in climate change scenarios and habitat suitability calculations.

Finally, the exposure of human populations to different levels of *Ae. albopictus* habitat suitability, i.e. referring to population and land area, is estimated using gridded population density data. For the recent past, we have used data available from the SEDAC service [60], while for the mid-century projections the population density was extracted using the medium-fertility scenario of the United Nations—Population Division [61,63]. Note that for the estimated population in the year 2050, changing patterns in human population (density) are not considered in this study. The results, summarized for six regions, are presented for the recent past and the mid-century projection in tables 2 and 3, respectively. These estimates indicate that approximately 2.4 billion individuals and an area of nearly 2 × 10^7^ km^2^ will be subject to high level *Ae. albopictus* habitat suitability (i.e. hsi greater than 70%) around the year 2050. Although the land area covered in the mid-century period is slightly less than in the recent past, the projected population growth together with the (habitat suitability) geographical distribution shift makes the mosquito threat even more important in future.

**Table 2.** Estimates of recent global population and surface area where the *Ae. albopictus* hsi exceeds 10, 35 and 70% based on 2005 population density for several regions.

	hsi > 10%		hsi > 35%		hsi > 70%	
year 2005	individuals (×10^6^)	area (×10^6^ km^2^)	individuals (×10^6^)	area (×10^6^ km^2^)	individuals (×10^6^)	area (×10^6^ km^2^)
region						
Europe	540	4.5	315	2.4	141	1.0
Africa	596	14.0	410	8.8	256	4.5
North America	271	3.7	185	2.5	105	1.4
South America	314	16.0	298	13.7	239	9.5
Asia	1760	8.0	1310	6.5	1040	5.1
Australia	14	2.3	12	1.2	7	0.5
global	3495	48.5	2530	35.1	1788	22.0

**Table 3.** Estimates of future global population and surface area where the *Ae. albopictus* hsi 10, 35 and 70% based on 2050 population density for several regions.

	hsi > 10%		hsi > 35%		hsi > 70%	
year 2050	individuals (×10^6^)	area (×10^6^ km^2^)	individuals (×10^6^)	area (×10^6^ km^2^)	individuals (×10^6^)	area (×10^6^ km^2^)
region						
Europe	606	5.2	340	2.4	174	1.0
Africa	1380	13.5	952	8.3	568	4.2
North America	464	5.3	330	3.3	173	1.8
South America	451	16.0	417	12.3	327	7.2
Asia	1810	7.2	1450	5.8	1150	4.7
Australia	20	2.6	18	1.3	10	0.4
global	4731	49.8	3507	33.4	2402	19.3

### (c) CMIP5 ensemble and robustness

Estimating uncertainties of climate model simulations is challenging [26,64]. Multi-model ensemble simulations have become a preferred procedure in climate assessments and evaluation of the simulation results, also applying various anthropogenic emission scenarios (IPCC SRES and RCPs). The models employed are continuously being improved in terms of simulating atmospheric and climatic processes in greater detail, thus enhancing the confidence level in terms of being more realistic [26], though not necessarily reducing overall uncertainty. It is argued by Knutti *et al.* [26] that judging climate model progress based on uncertainties is a rather strict criterion. Following Knutti *et al.* [26], we assess the performance of the CMIP5 and EMAC models for the geographic regions of relevance to our study using the robustness measure *R*. This metric, which is based on the signal-to-noise ratio and the ranked probability skill score [38], takes into account the sign and magnitude of the difference, variability and inter-model spread. Robustness with a value *R* = 1 suggests that the agreement between the models is perfect and the confidence is greatest, while a value of *R* = 0 implies no relative skill or confidence at all. We should stress that the choice of the cut-offs for *R* used to demonstrate the model agreement is rather subjective, but nevertheless the higher the threshold value the greater the confidence [26].

**Figure 10.**
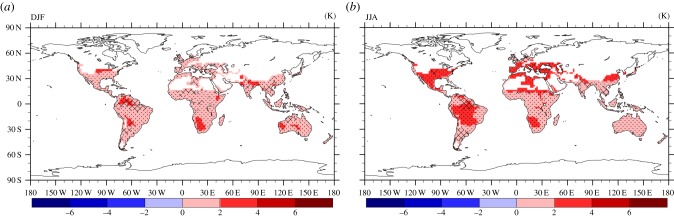
Multi-model mean and robustness (dots (*R* ≥ 0.75) and cross-hatching (0.5 < *R* < 0.75)) for the change in near-surface temperature in DJF (*a*) and JJA (*b*) for 2045–2054 compared with 2000–2009.

**Figure 11.**
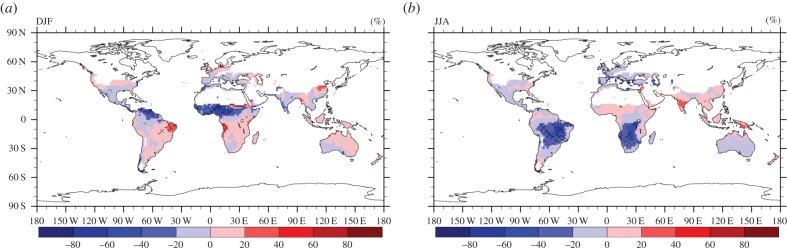
Multi-model mean and robustness (dots (*R* ≥ 0.5) and cross-hatching (0.2 < *R* < 0.5)) for the relative (%) precipitation (flux) change in DJF (*a*) and JJA (*b*) for 2045–2054 compared with 2000–2009.

We computed *R* for both the near-surface temperature and precipitation fields and the results are presented in figures 10 and 11, respectively. Figure 10 depicts the mean boreal winter (DJF) and summer (JJA) near-surface temperature change, comparing the simulation periods 2000–2009 and 2045–2054. Robustness larger than 0.75 is shown with dots, while cross-hatching represents robustness between 0.5 and 0.75. Likewise, figure 11 shows the mean boreal winter (DJF) and summer (JJA) relative precipitation change, where robustness greater than 0.5 is displayed with dots, and robustness between 0.2 and 0.5 by cross-hatching. For the near-surface temperature, the dominant value of *R* > 0.75 indicates a rather good model agreement implying a high confidence level in the multi-model results. On the contrary, for precipitation we find a very low level of agreement between the models, especially over land. This is to some degree expected as climate models, especially at coarse grid resolution, are less skilled in simulating precipitation patterns and intensity, in particular by deep convection, being represented by sub-grid scale parametrizations.

**Figure 12.**
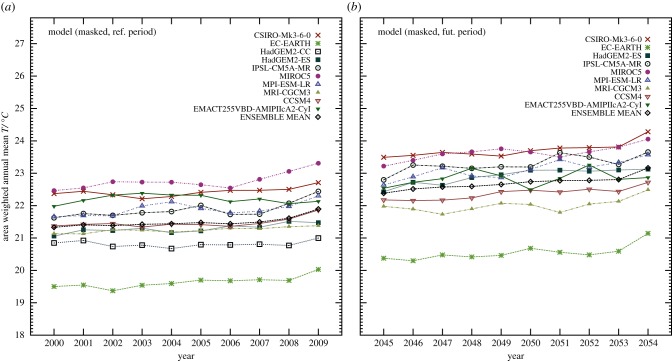
Area-weighted mean (near-surface) temperature (*a*) for the recent period 2000–2009 for the CMIP5 ensemble and the interpolated EMAC T255 result, and (*b*) for the future period 2045–2054. For the comparisons we have masked land areas where the hsi is less than 10%. For a similar comparison that includes the unmasked global values, refer to the electronic supplementary material, figure S23.

To get an impression of the simulated climate change by EMAC at T255 resolution under the SRES-A2 scenario relative to the CMIP5 models under the RCP8.5 scenario, figure 12*a* shows the area-weighted mean temperatures for the recent period 2000–2009 and figure 12*b* for the future period 2045–2054. The oceans have been excluded and the land areas with an hsi less than 10% have also been masked. Both EMAC outcomes seem to be consistent with the CMIP5 models, especially for the simulated future projections where our model is closest to the ensemble average. Notably in the future period, our model results indicate larger inter-annual variability, possibly related to the higher resolution of the simulations.

### (d) Habitat suitability based on CMIP5

In this section, we study the effect of grid size and test the representativeness of our calculations in view of the most recent climate projections. We emphasize that from the available daily datasets in CMIP5 not all models report the RH. Thus for a fair comparison, the corresponding criteria of the vector distribution model were switched off in extracting the habitat suitability index from the EMAC T255 data.

**Figure 13.**
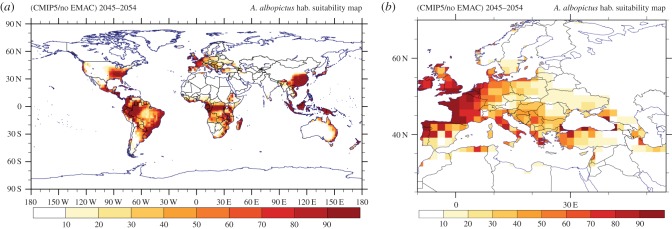
CMIP5 multi-model ensemble (mean) habitat suitability over the projected (RCP8.5 concentration pathway) period 2045–2054. The average does not include EMAC T255 simulation results.

For the projected period between 2045 and 2054, the habitat suitability is illustrated in figure 13. On a global scale the pattern of potentially suitable areas supporting the establishment of the vector is similar to that calculated by EMAC (figures 6 and 7). The Pearson spatial (grid-space) correlation coefficient for the projected period between CMIP5 (RCP8.5) and the regridded EMAC (SRES-A2) indicates good agreement (*r* = 0.85 [99.9% CI, *p* < 0.001]). By excluding the RH variable from the multi-criteria decision vector model, the corresponding habitat suitability shows a slightly higher correlation (*r* = 0.86 [99.9% CI, *p* < 0.001]). Although the global maps (figures 6*b* and 13*a*) appear to be similar, the regional maps (figures 7*b* and 13*b*) illustrate that the coarse grid global model ensemble misses many details that can be relevant for vector control policies. For example, the degree to which coastal areas and islands are affected, especially in subtropical regions, is more realistically represented at high resolution, which is illustrated by comparing the Mediterranean regions in figures 7*b* and 13*b*.

## 4. Discussion and conclusion

*Aedes albopictus* is a public health threat owing to its environmental adjustability and its capability to transmit the pathogens that cause dengue fever, chikungunya infection, West Nile fever and potentially other diseases. The global distribution of this dangerous vector has shown considerable changes during the past decades. As the body temperature of *Ae. albopictus* is regulated by environmental conditions, climate change potentially influences its distribution. To assess the present and future geographical regions that can provide suitable habitat conditions, we employed a multi-criteria vector distribution model based on seven meteorological variables related to temperature, precipitation and RH [3,5,39,40]. The seven predictor variables included in the multi-decision criteria vector distribution model are represented by fuzzy-logic membership functions, which signify the climatic suitability on a 0–100 scale. The meteorological fields have been averaged for 10-year periods, one for the recent past (2000–2009) and one for the middle of the century (2045–2054), computed with a comparatively high-resolution (50 km) version of the EMAC global climate model. We show that this resolution is particularly helpful to represent topographically pronounced and coastal areas, the latter typically associated with high population density worldwide, whereas global climate simulations are usually performed at much coarser resolution. The 50 km grid scale is also close to that employed in previous regional studies, e.g. of Fischer *et al.* [12,14] and Caminade *et al.* [11] who focused on Europe, for which we find many similarities with our results.

For example, for the recent past all studies indicate a relatively large degree of habitat suitability in the western Iberian Peninsula, France, Italy and the western Balkan Peninsula and a northward spread into Europe in future. However, the results of Caminade *et al.* [11], who also followed a process-based approach, seem slightly closer to our results than those reported by Fischer *et al.* [12,14], using a statistically based niche modelling approach. For future projections, the three-variable model used by Caminade *et al.* [11] predicts larger areas in Central and Eastern Europe as suitable for the mosquito than our seven-variable model, possibly because our model introduces additional constraints and/or because our climate simulations imposed boundary conditions from a different future emissions scenario. In the electronic supplementary material, figures S20 and S21 (left columns), we present the habitat suitability maps referring to Europe, estimated using a three-variable model driven by annual rainfall, January minimum and summer maximum temperatures [10,11]. Absolute differences between the three- and seven-variable models, for both simulation periods, are also provided in the electronic supplementary material, figure S22. Moreover, comparing the results of Fischer *et al.* [14] with our results, a noticeable difference is evident regarding the habitat suitability predictions for the British Isles and Bretagne (France); for the recent period they report a moderate climatic suitability while it is more pronounced in our results. For the mid-century projections, Fischer *et al.* [14] indicate that the aforementioned areas become ‘increasingly suitable’, based on the SRES-A1B (intermediate) emissions scenario. The overall pattern differences may be attributed to the different assumptions followed by the two (species distribution) modelling approaches, whereas the hsi differences may again be attributed to the climate change scenarios being employed.

For the recent past simulations (2000–2009), we forced the EMAC model with AMIP-IIc sea surface temperature and sea-ice assimilation data, whereas for the future (2054–2054), the climate projection is based on the SRES-A2 greenhouse gas emission estimates, representing a relatively pessimistic scenario (strong warming), roughly equivalent to the more contemporary RCP8.5 pathway. The recent past simulation has been evaluated against EI meteorological re-analysis data over the same period, indicating a very high grid–space correlation for near-surface temperature though a slight warm bias of our model in the regions where suitable habitat conditions (i.e. hsi > 10%) are expected. For precipitation we obtain lower correlation coefficients, though nevertheless reasonable agreement. For the future scenario, we tested the model robustness (*R*) based on the recent CMIP5 climate model output of eight additional climate models forced by the RCP8.5 pathway. We find a high degree of model consistency and good agreement with our calculations for the period 2045–2054 for the near-surface temperature (*R* > 0.75), while the robustness for precipitation is much less (*R* < 0.2).

Our habitat suitability calculations indicate highly favourable conditions for *Ae. albopictus* in most of the wet tropical regions and somewhat reduced but nevertheless very suitable conditions in the subtropical parts of Brazil, the southeastern United States, southern Africa, Madagascar, Southeastern China and the northern part of the Mediterranean basin. The most northerly occurrence (subsequent to introduction of the mosquito) may be expected in Western Europe. Our calculations appear qualitatively consistent with the observed presence of *Ae. albopictus*. A more systematic evaluation of our model results would be possible if the observational database were to improve, i.e. if existing data were to become publicly available. Based on our climate simulations for the period 2045–2054, a poleward shift of the suitable habitat conditions may be expected. A significant increase of habitat suitability is projected to occur in eastern Brazil, the eastern United States, Western and Central Europe, and Eastern China, to a large degree related to increasing near-surface temperatures in winter. On the other hand, significant reductions are projected for northern South America, Southern Europe, Central Africa, Madagascar and Southeast Asia, where (summer) temperatures can become too high. In general, it seems that environmental conditions in the tropics, where *Ae. albopictus* is native become less suitable, whereas other regions become more predisposed to mosquito invasion, allowing the species to compensate for the loss of territory. Furthermore, combining the simulated hsi above 70% and population projections (for 2050), we estimate that 2.4 billion people will live in areas that are climatically favourable for *Ae. albopictus*.

For future work, we recommend a suitable weighting scale on the fuzzy-logic membership functions based on expert judgement [54,56], accounting for the relative importance of environmental factors for the survival of *Ae. albopictus*, and additional laboratory work and data collection to refine the break-points and rates of change relating to the degree of suitability of each meteorological (predictor) variable. It is also useful to consider the sensitivity of the vector distribution model to the variabilities associated with the weight factors assigned by the field experts, and to the uncertainties related to the predictor variables, respectively. It should be stressed that another important aspect, not considered in this study, is the role of globalization. This has a direct link to the geographical dispersal of *Ae. albopictus*. Owing to the intense global transportation and in conjunction with climate change [65], seaports and airports may act as potential gateways for the vector species and their associated pathogens, which could easily be transmitted from their endemic or epidemic areas into these, usually densely populated, urban localities. We suggest the refined representation of urban areas (including harbours and major airports) in climate modelling, since *Ae. albopictus* is a container-breeder living in close association with humans, and urban environments can provide the micro-climatic conditions that influence its habitat suitability and could be important for its enhanced spread.

## Supplementary Material

Supplementary material

## Supplementary Material

CABI data (presence locations at country/state level) for Ae. albopictus

## Endnotes

1 The suitability index,  is expressed relative to the maximum suitability score  For example, a suitability score of 60 is equivalent to a suitability index (si) of 60%.

2 For the *r*-test comparison statistics, we have not excluded areas with a habitat suitability index less than 10%.
