# Supplementary material for: Present and future projections of habitat suitability of the Asian tiger mosquito, a vector of viral pathogens, from global climate simulation
Source: Philos Trans R Soc Lond B Biol Sci. 2015 Apr 5;370(1665):20130554. doi: 10.1098/rstb.2013.0554 (PMC4342960; doi:10.1098/rstb.2013.0554)
Supplement: Supplementary material [file rstb20130554supp1.pdf]

Present and Future Projections of habitat suitability of the Asian Tiger Mosquito, a vector of viral pathogens, from global climate simulations

Y. Proestos[1], G. K. Christophides[3], K. Ergüler[1], M. Tanarhte[2], J. Waldock[1,3] and J. Lelieveld[1,2]

[1]The Cyprus Institute, Cyprus
[2]Max Planck Institute for Chemistry, Germany
[3]Imperial College, UK

July 18, 2014

Electronic supplementary material

This electronic supplementary material includes additional figures and information that complements the manuscript.

1 EMAC climatic fields used in the vector distribution model

1.1 Recent past: 2000-2009

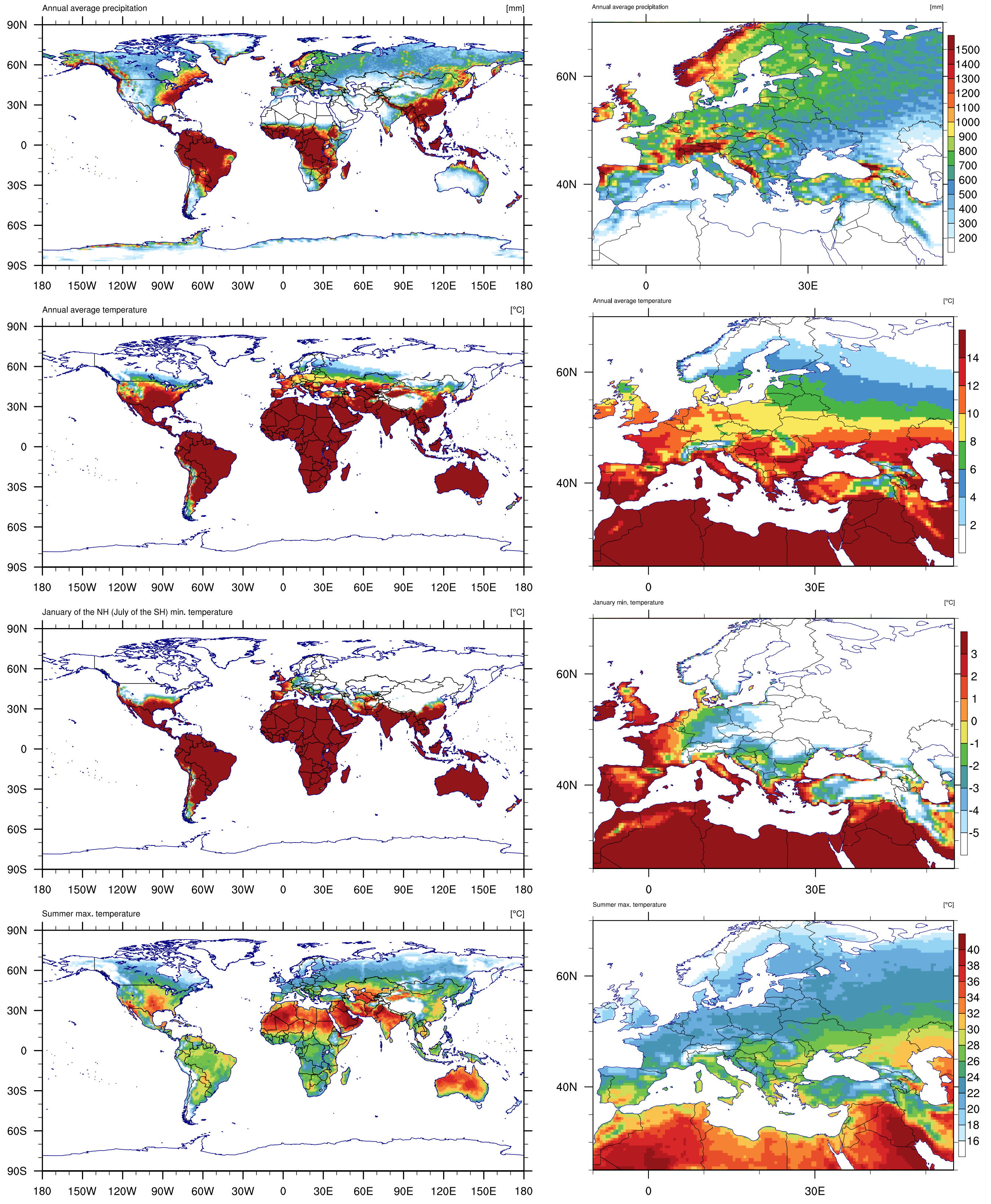

Figure S1: Global and European climatologies of the factors applied in the vector distribution model for the period 2000 to 2009.

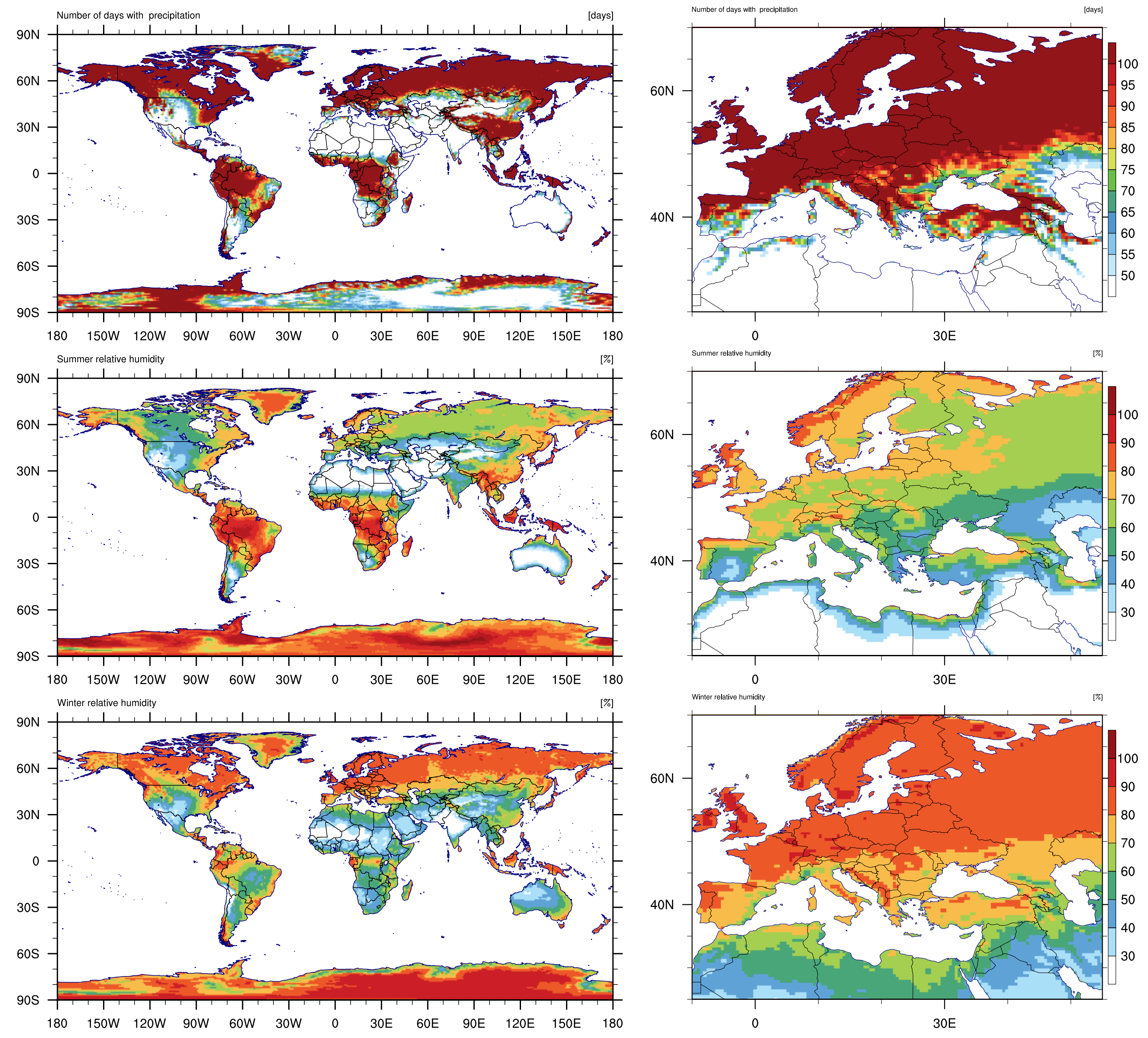

Figure S2: Global and European climatologies of the factors applied in the vector distribution model for the period 2000 to 2009.

1.2 Mid-century projections: 2045-2054

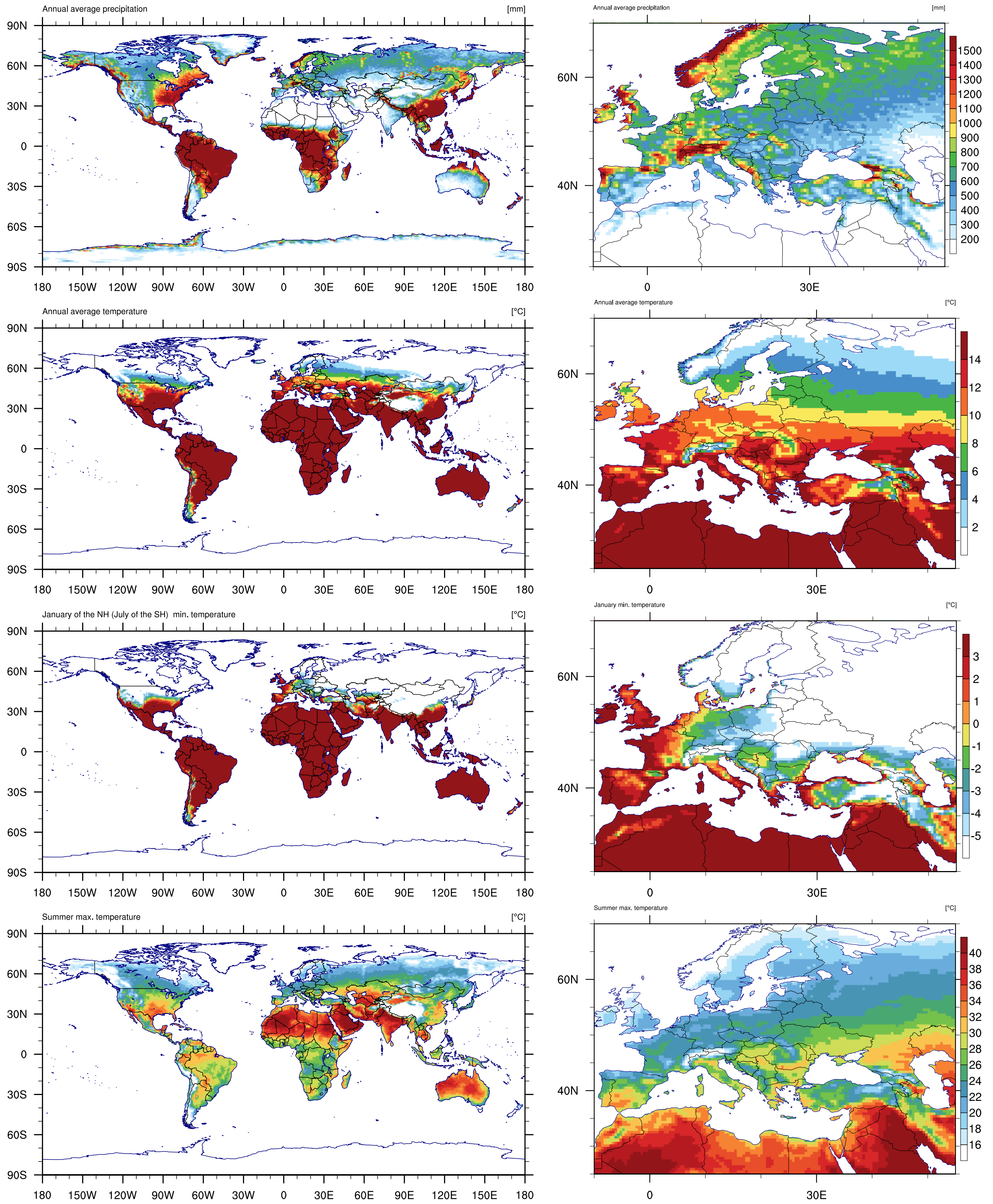

Figure S3: Global and European climatologies of the factors applied in the vector distribution model for the period 2045 to 2054.

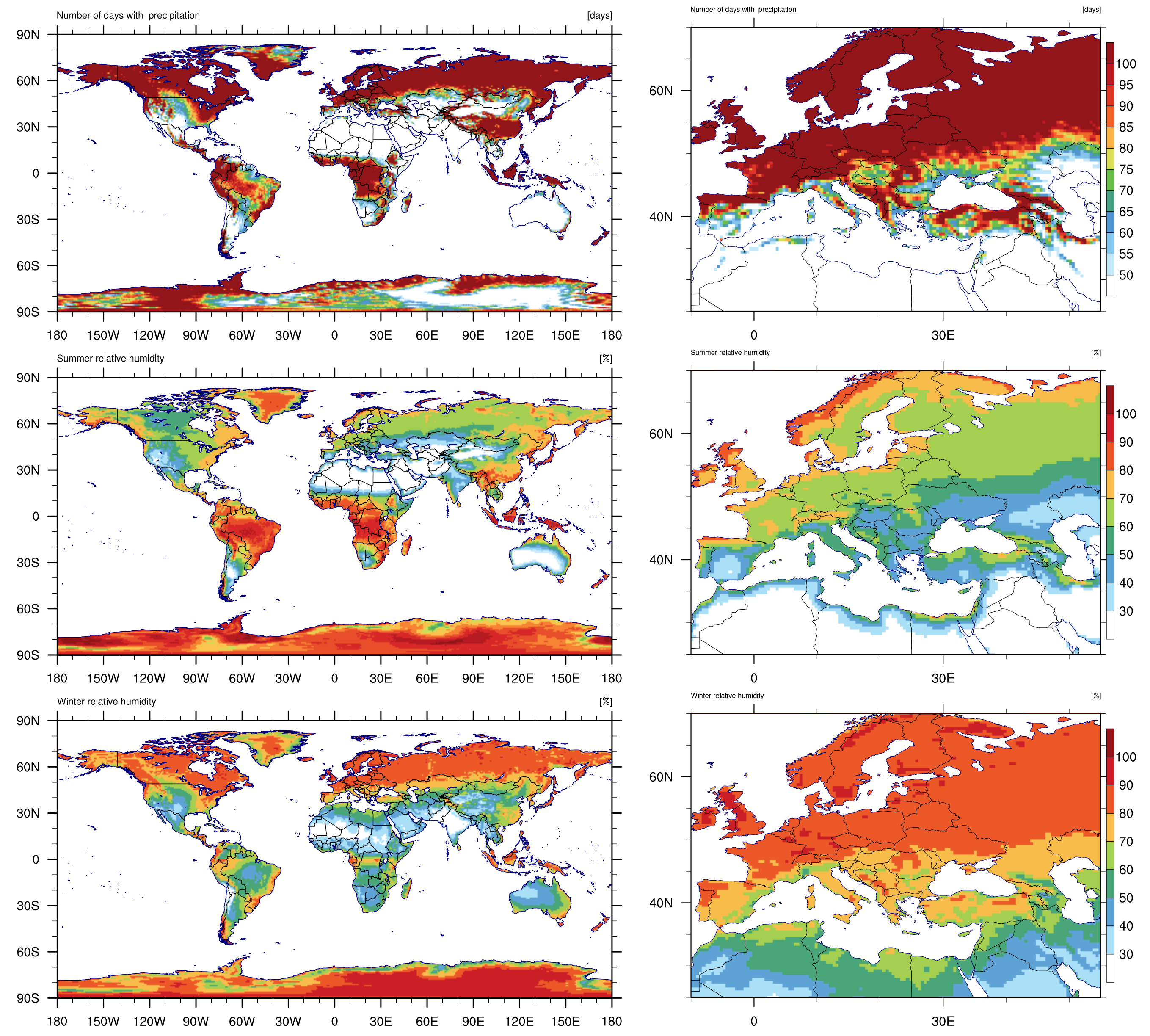

Figure S4: Global and European climatologies of the factors applied in the vector distribution model for the period 2045 to 2054.

2 Suitability scores-EMAC

2.1 Recent past: 2000-2009

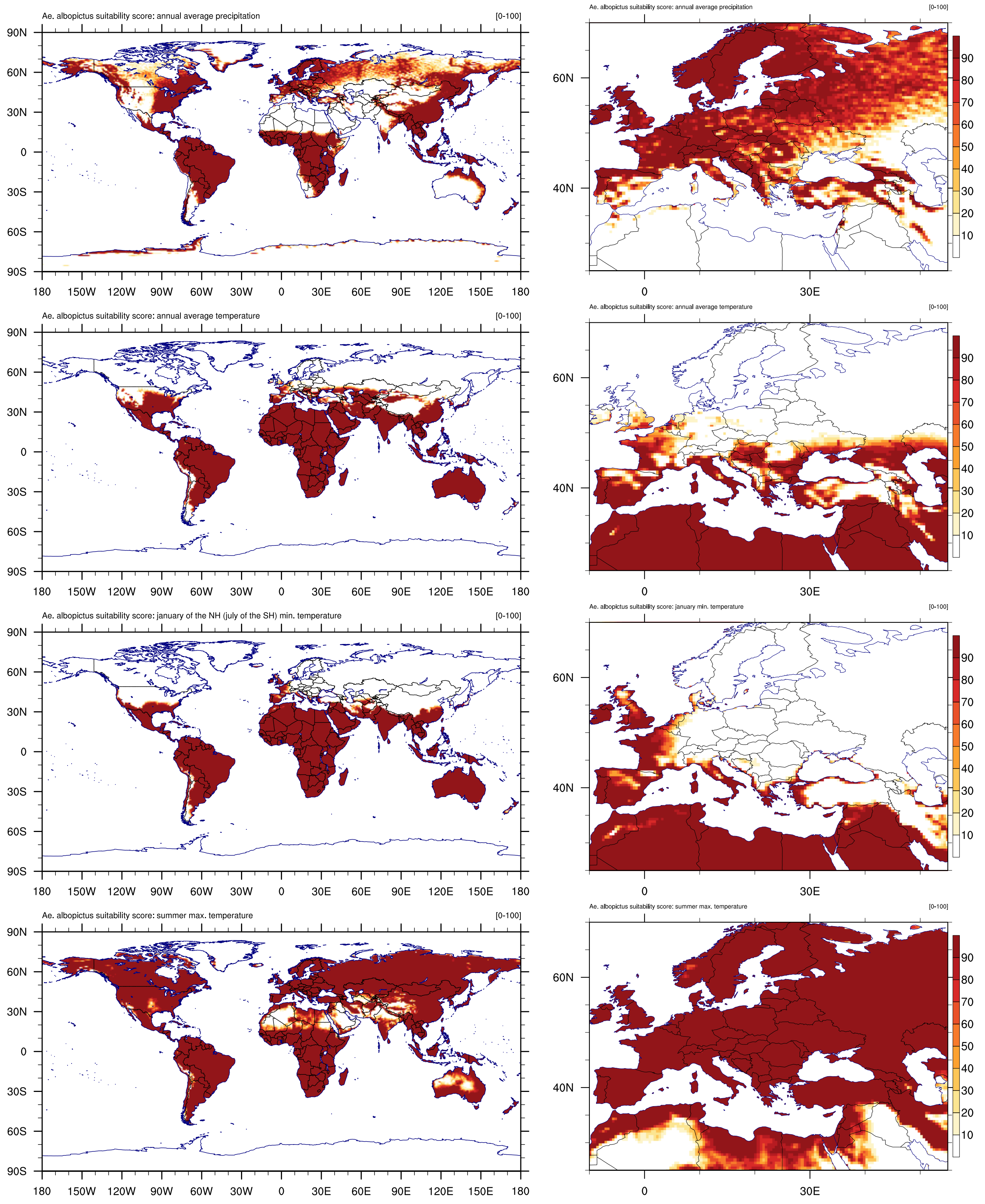

Figure S5: Global and European suitability scores for each of the factors used in the vector distribution model for the period 2000 to 2009.

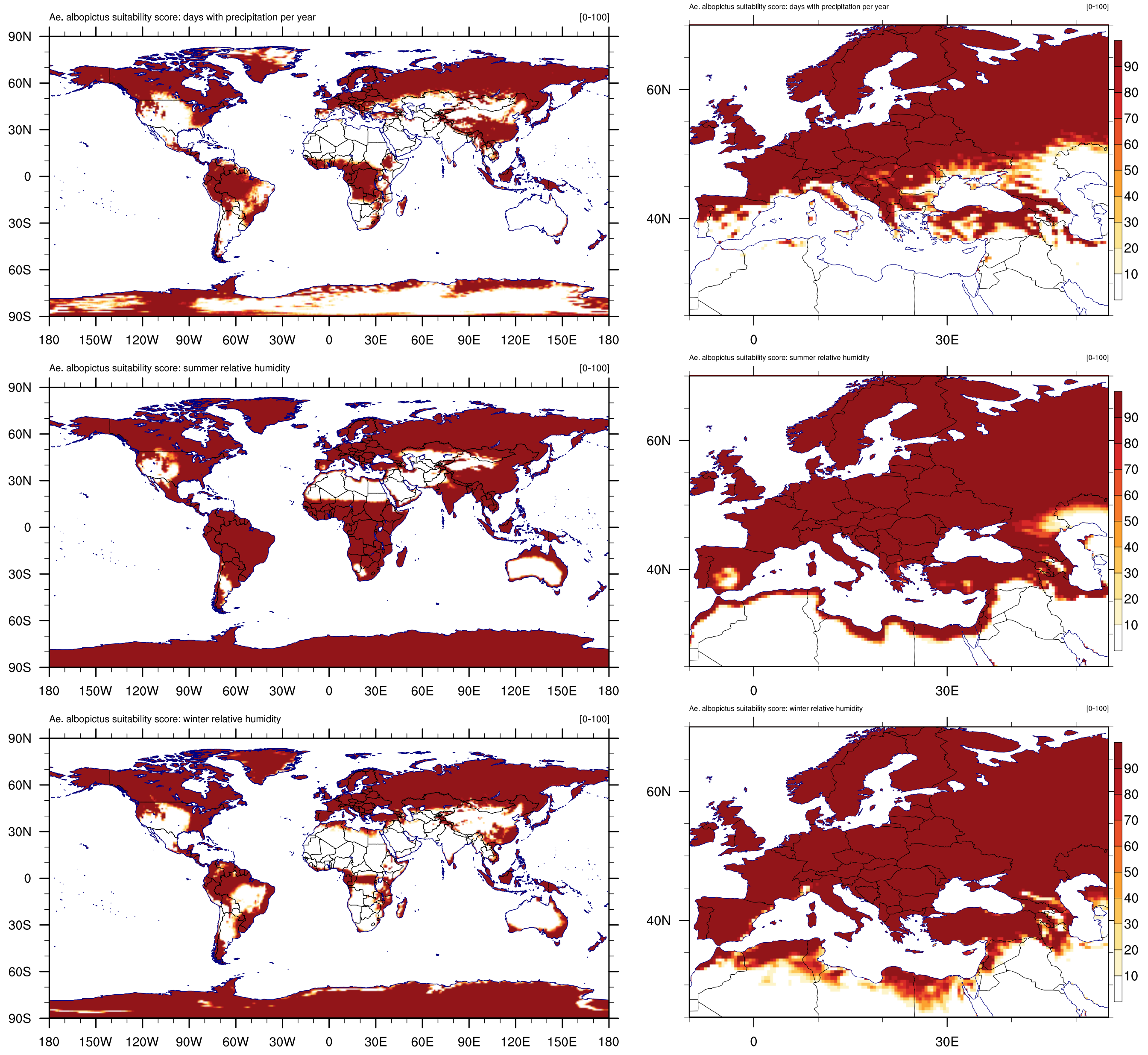

Figure S6: Global and European suitability scores for each of the factors used in the vector distribution model for the period 2000 to 2009.

2.2 Mid-century projections: 2045-2054

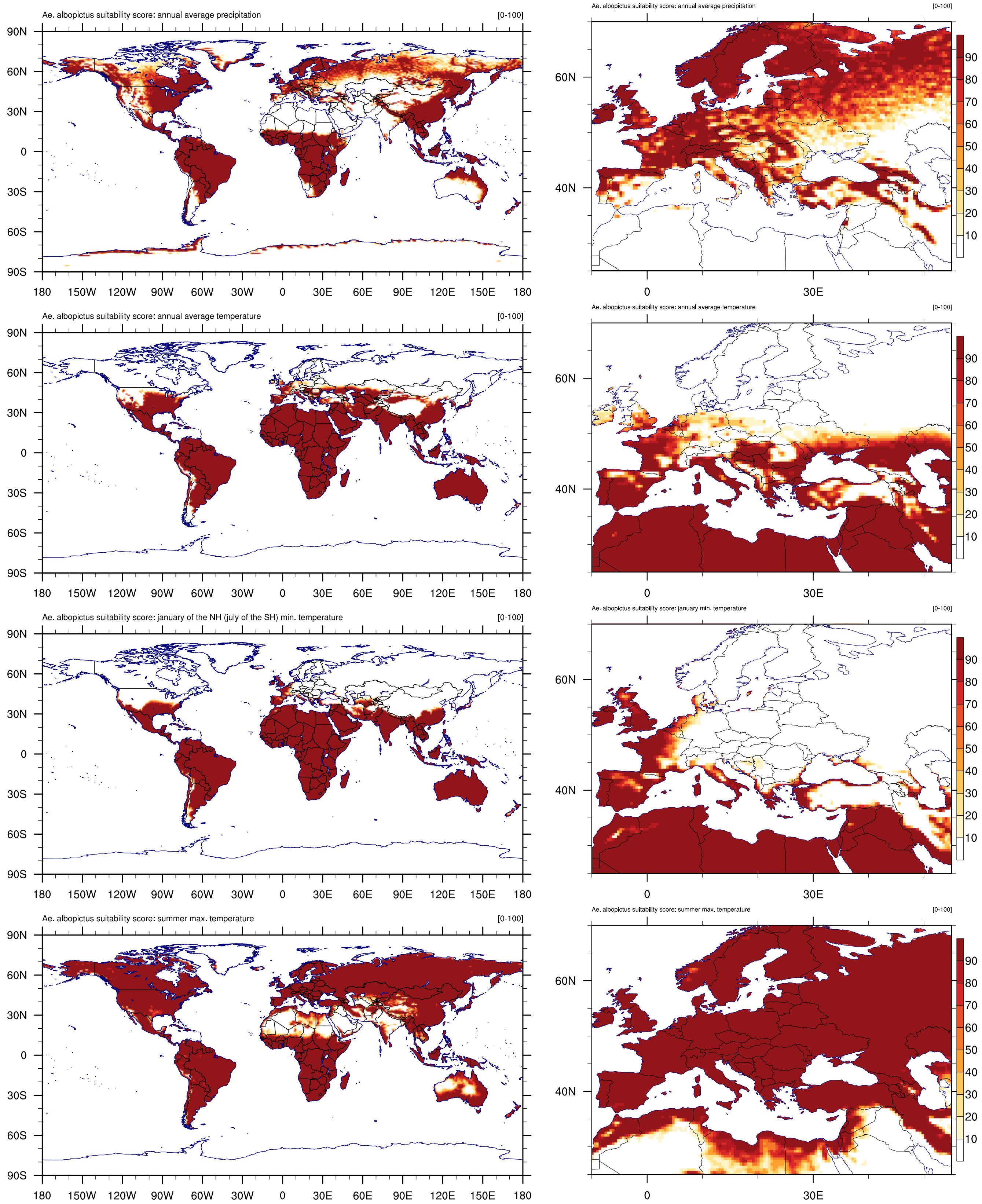

Figure S7: Global and European suitability scores for each of the factors used in the vector distribution model for the period 2045 to 2054.

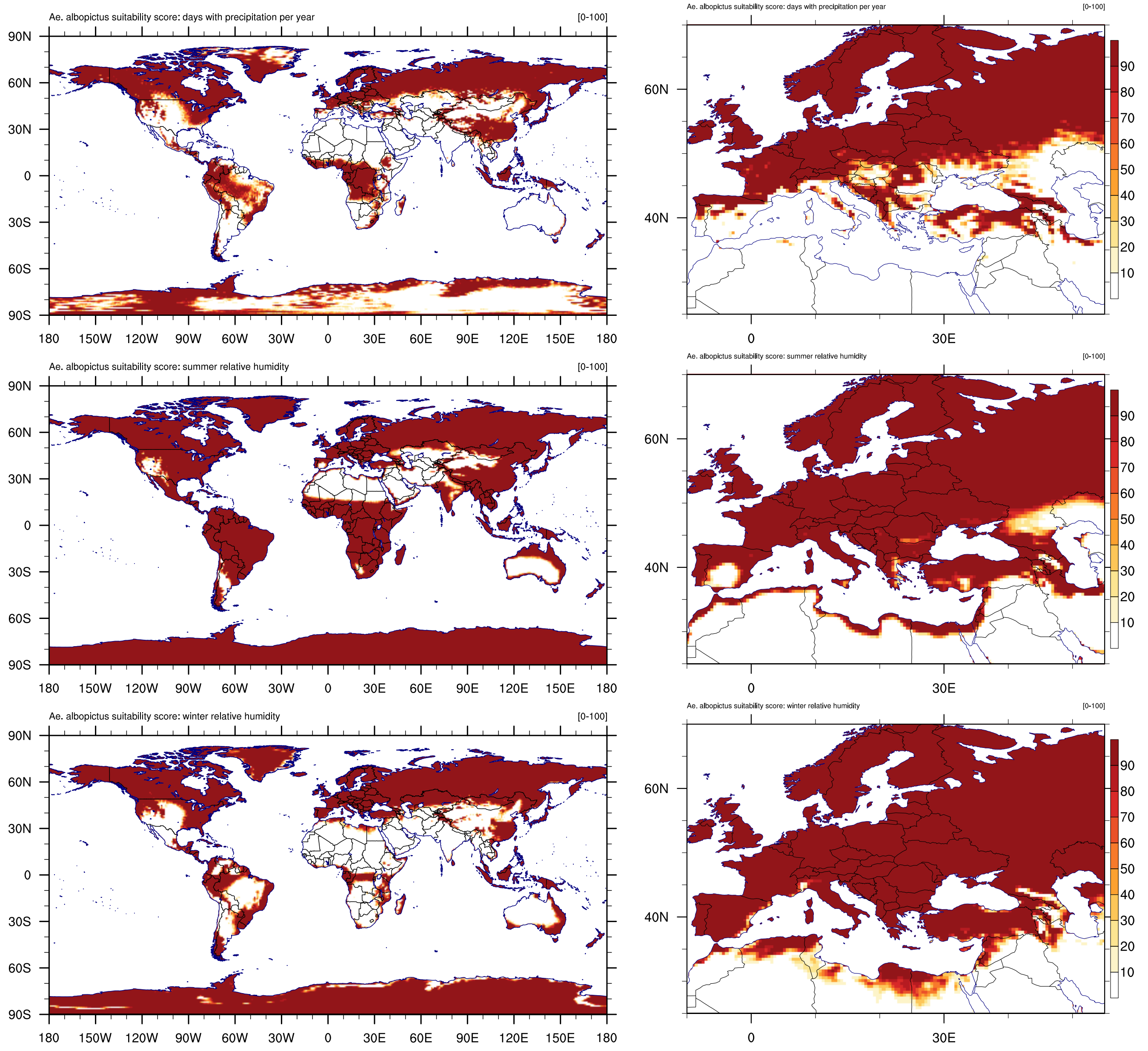

Figure S8: Global and European suitability scores for each of the factors used in the vector distribution model for the period 2045 to 2054.

3 Habitat suitability per region

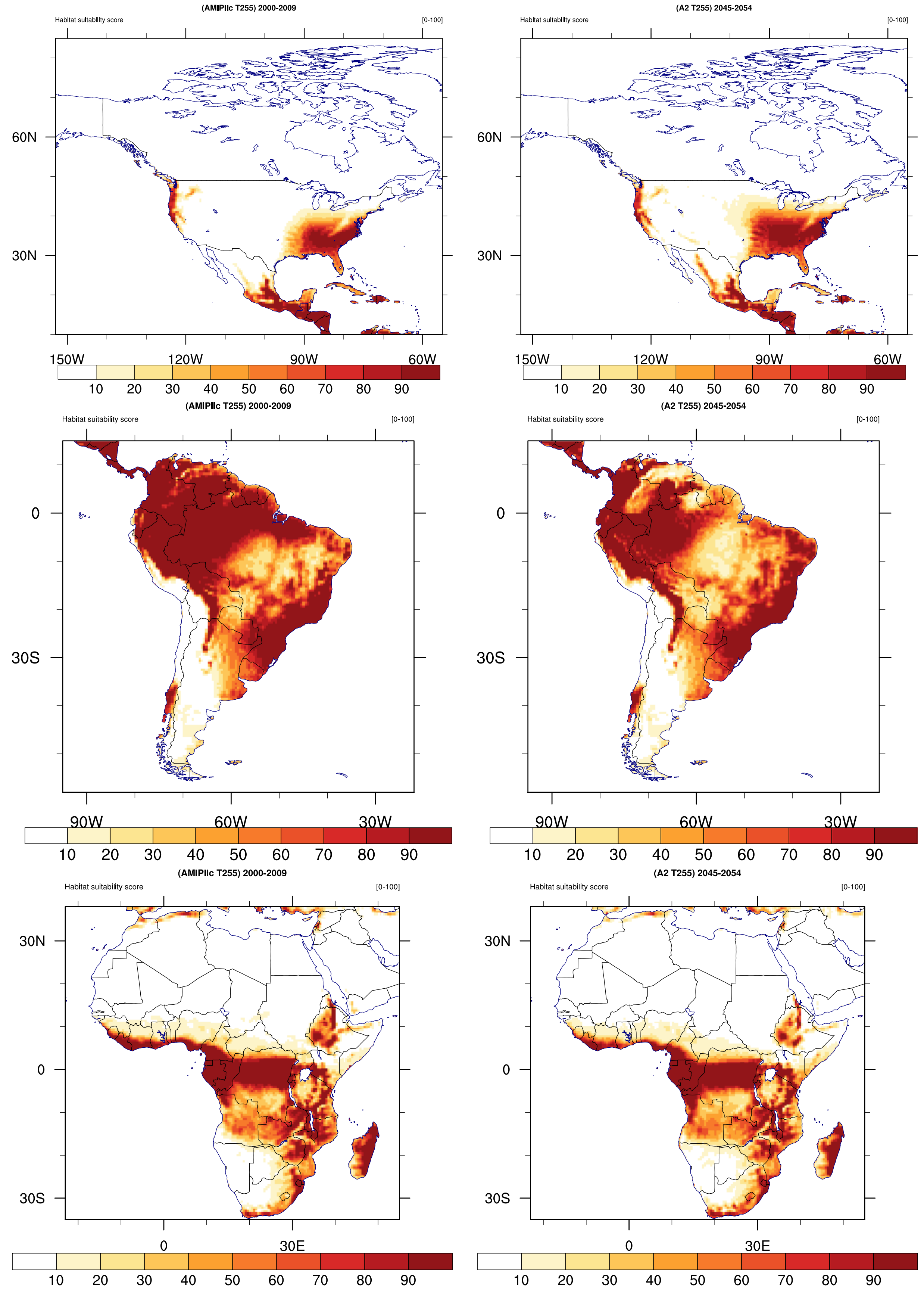

Figure S9: EMAC simulated habitat suitability for both the (left column) recent past (2000-2009) and (right column) mid-century projections (2045-2054).

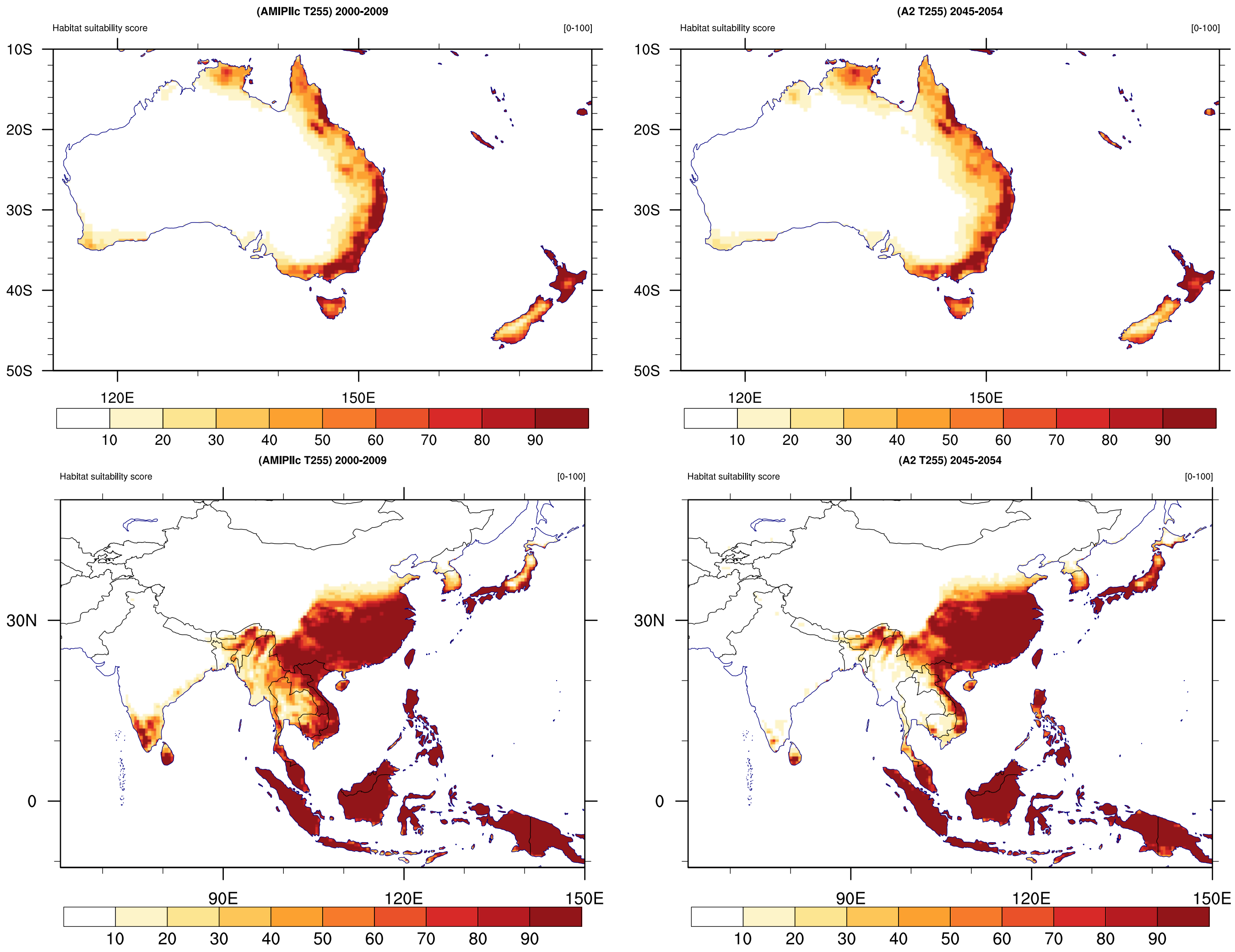

Figure S10: EMAC simulated habitat suitability for both the (left column) recent past (2000-2009) and (right column) mid-century projections (2045-2054).

3.1 Habitat suitability changes per region

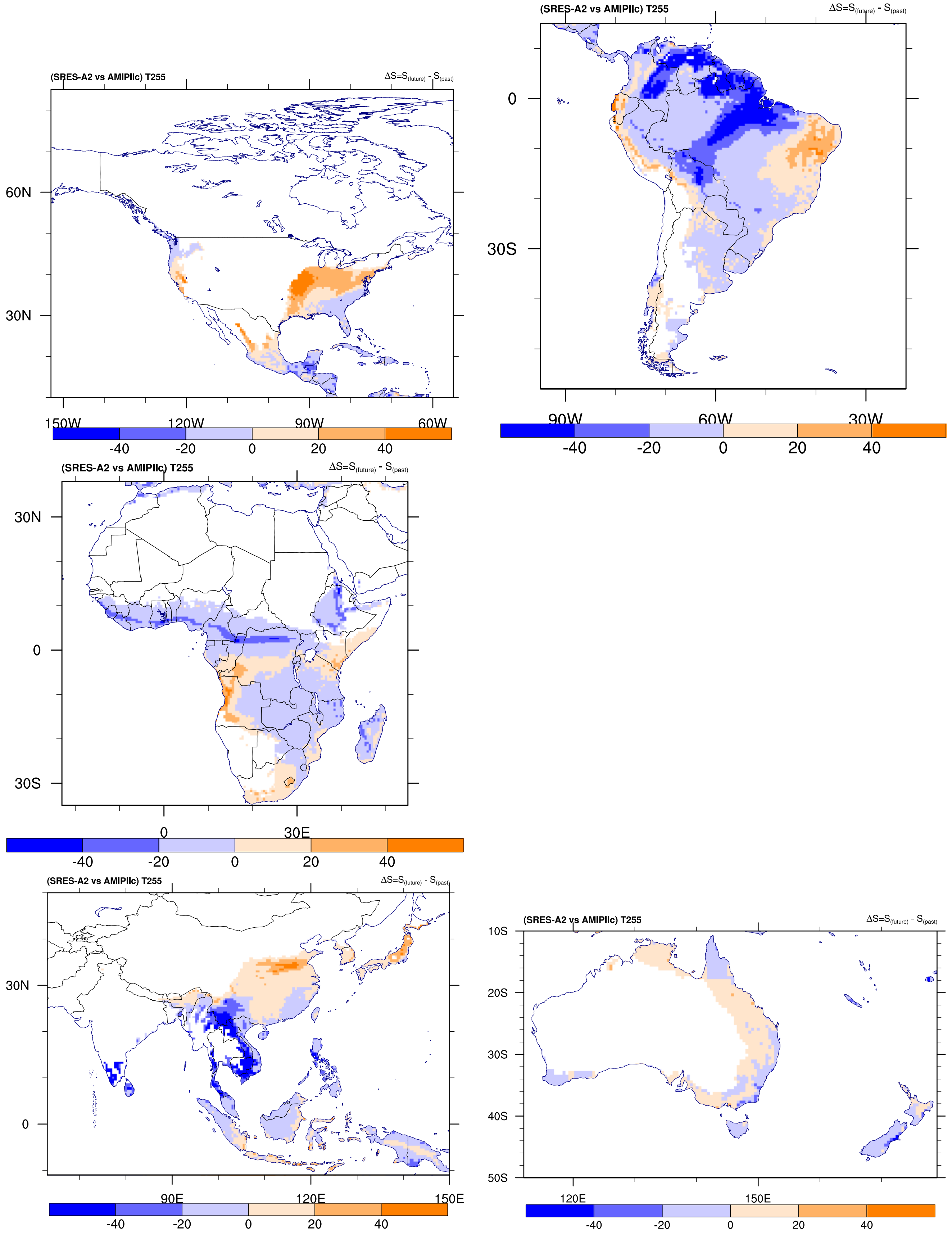

Figure S11: Differences in habitat suitability between the (left column) recent past period (2000-2009) and (right column) mid-century projections (2045-2054). Areas with an hsi lower than 10% have been excluded.

4 Comparing EMAC near surface temperature and precipitation to ERA-Interim

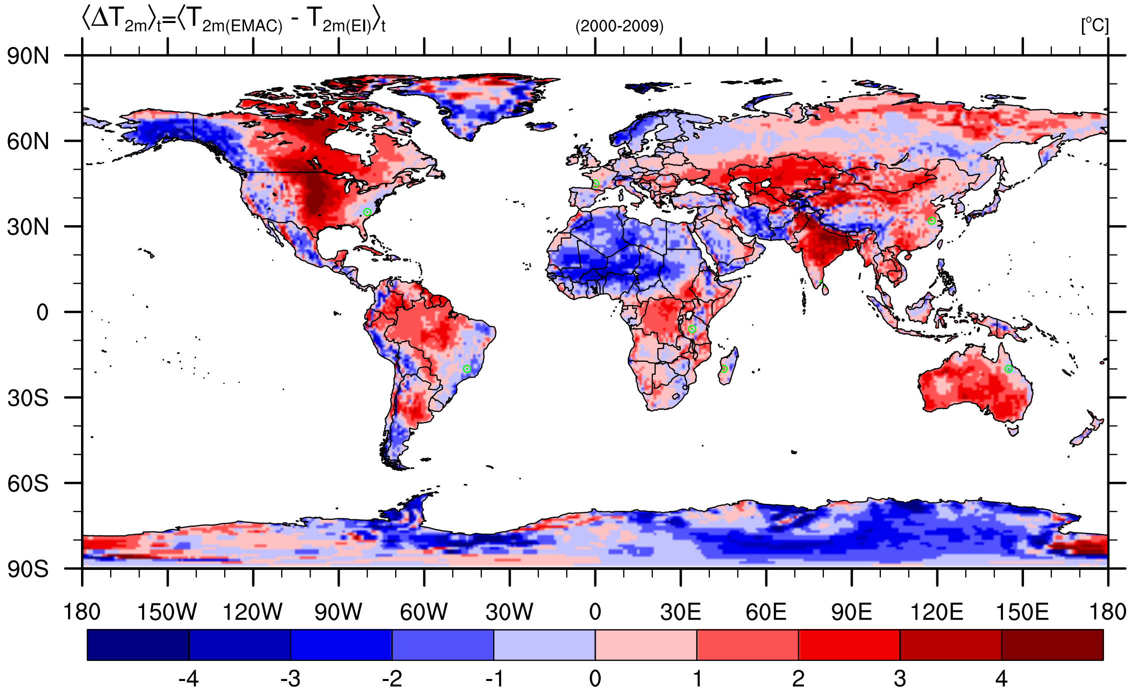

Figure S12: Comparing the EI data with the model simulation over the period 2000-2009. Mean near surface temperature difference. (b) Average precipitation difference.

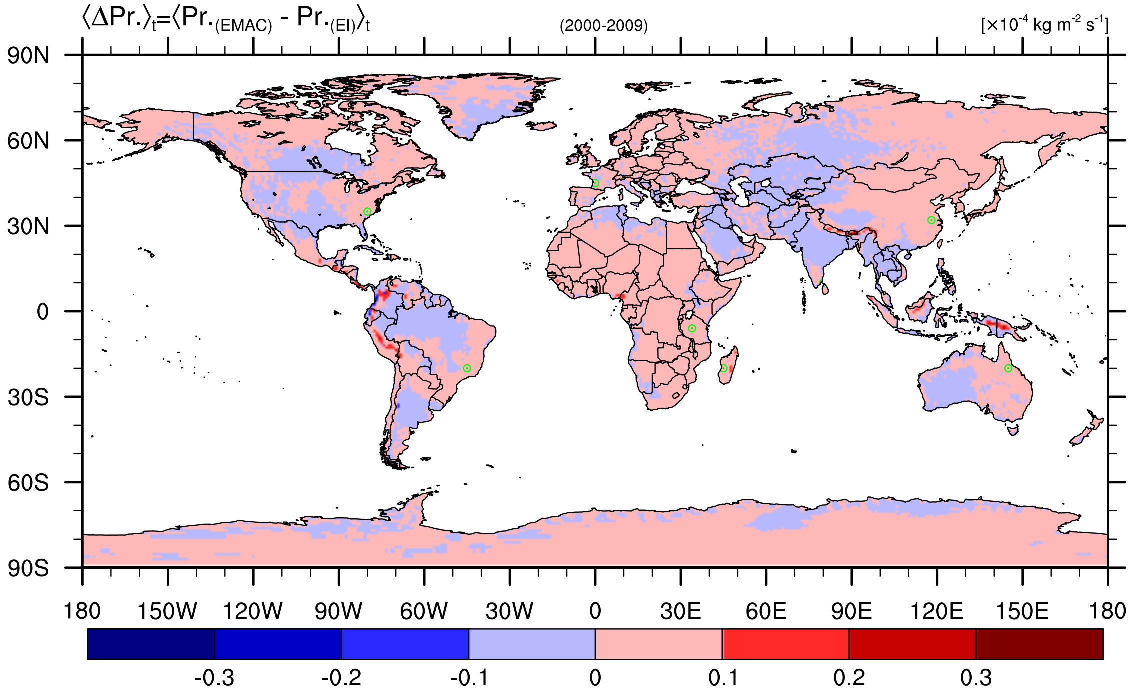

Figure S13: Comparing the EI data with the model simulation over the period 2000-2009. Average precipitation difference.

Pearsons r-test introduced in the manuscript to highlight the degree of linearity between the EMAC and ERA-Interim datasets (for near surface temperature and precipitation fields, respectively). In order to examine the actual linear relation between the two datasets we plot EMAC against EI data providing the corresponding regression lines.

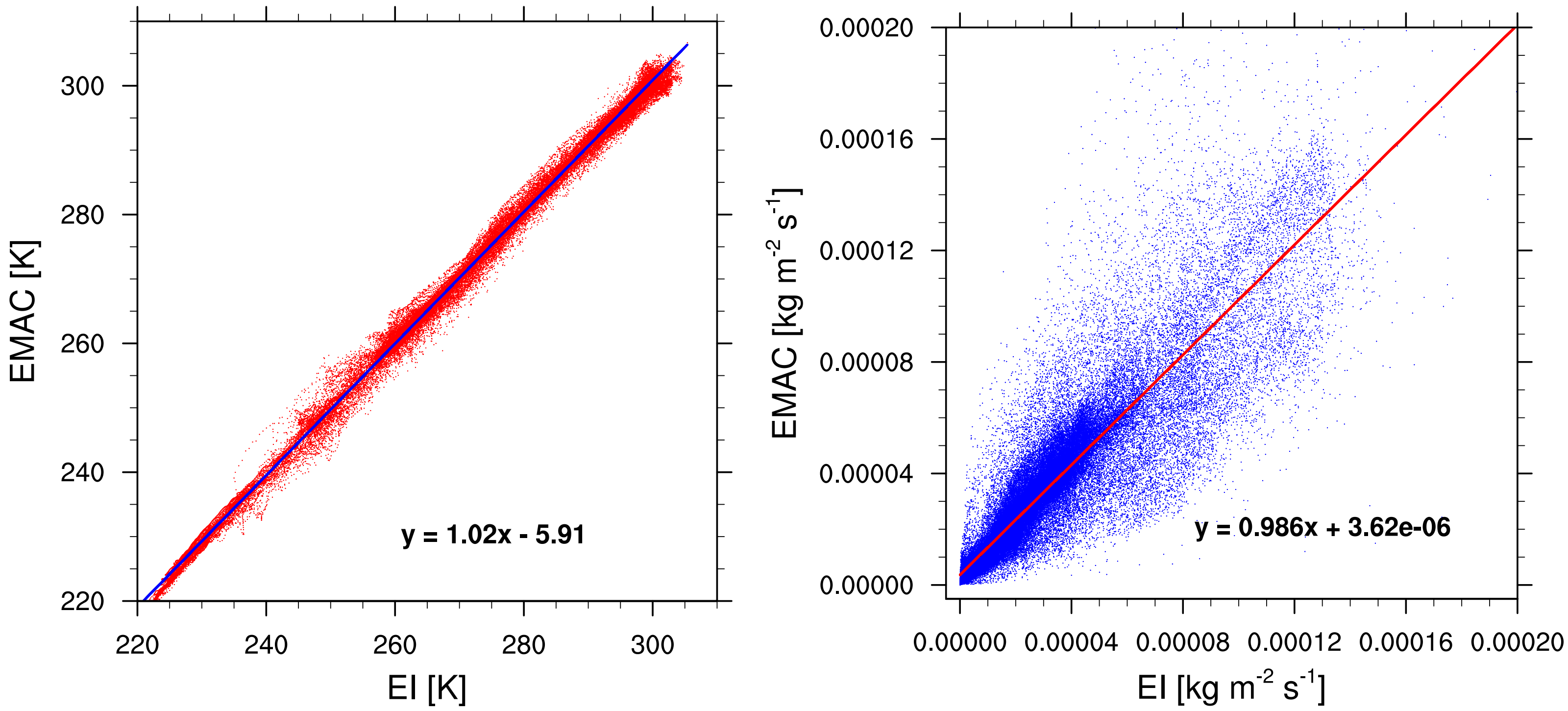

Figure S14: EMAC data plotted against EI data along with linear regression fits for near surface temperature (left) and precipitation (right).

5 ERA-Interim climatic fields–period 2000 to 2009

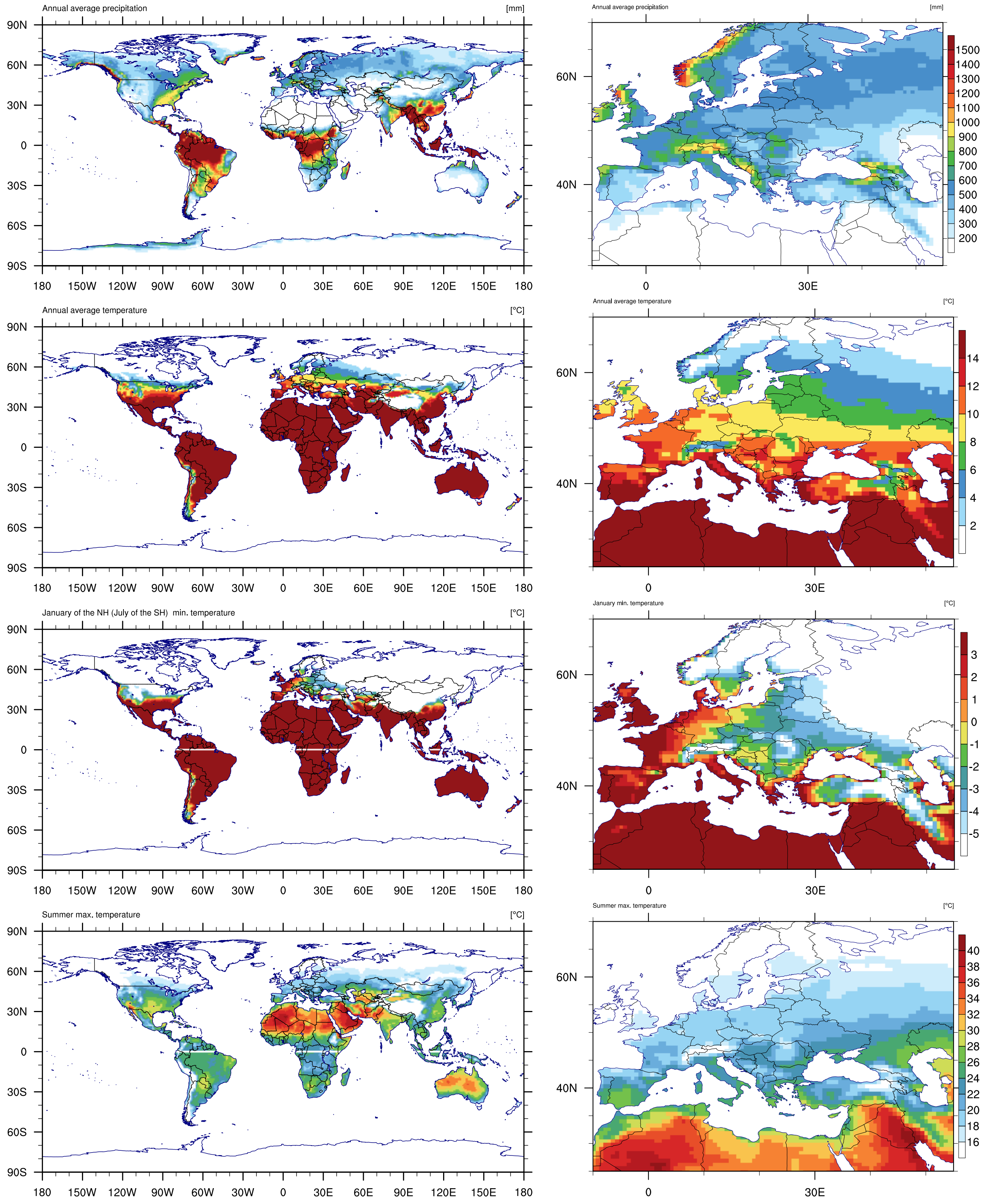

Figure S15: Climatologies of the factors applied in the vector distribution model for the period 2000 to 2009, using ERA-Interim data.

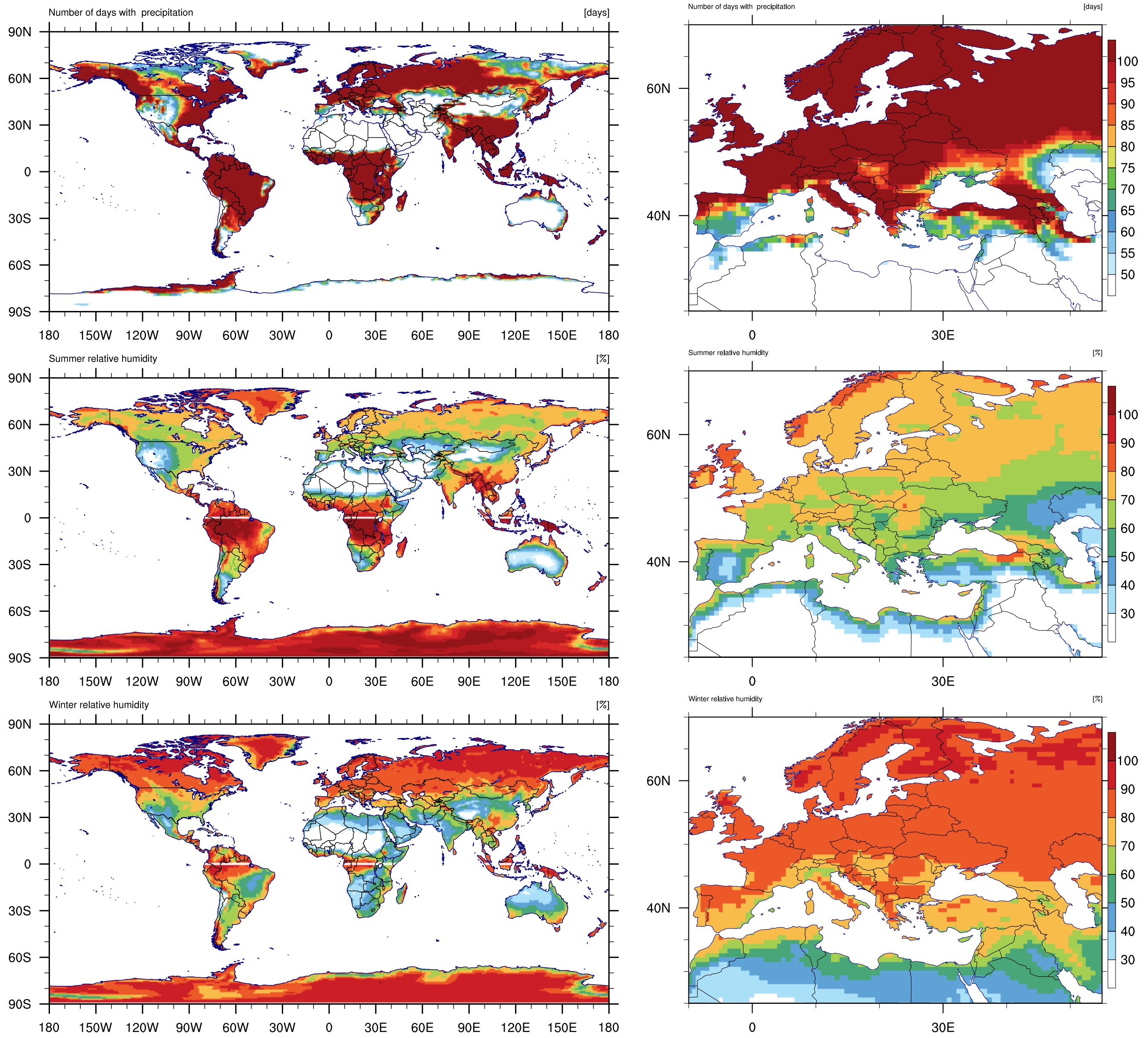

Figure S16: Climatologies of the factors applied in the vector distribution model for the period 2000 to 2009, using ERA-Interim data.

6 Suitability scores–ERA-Interim

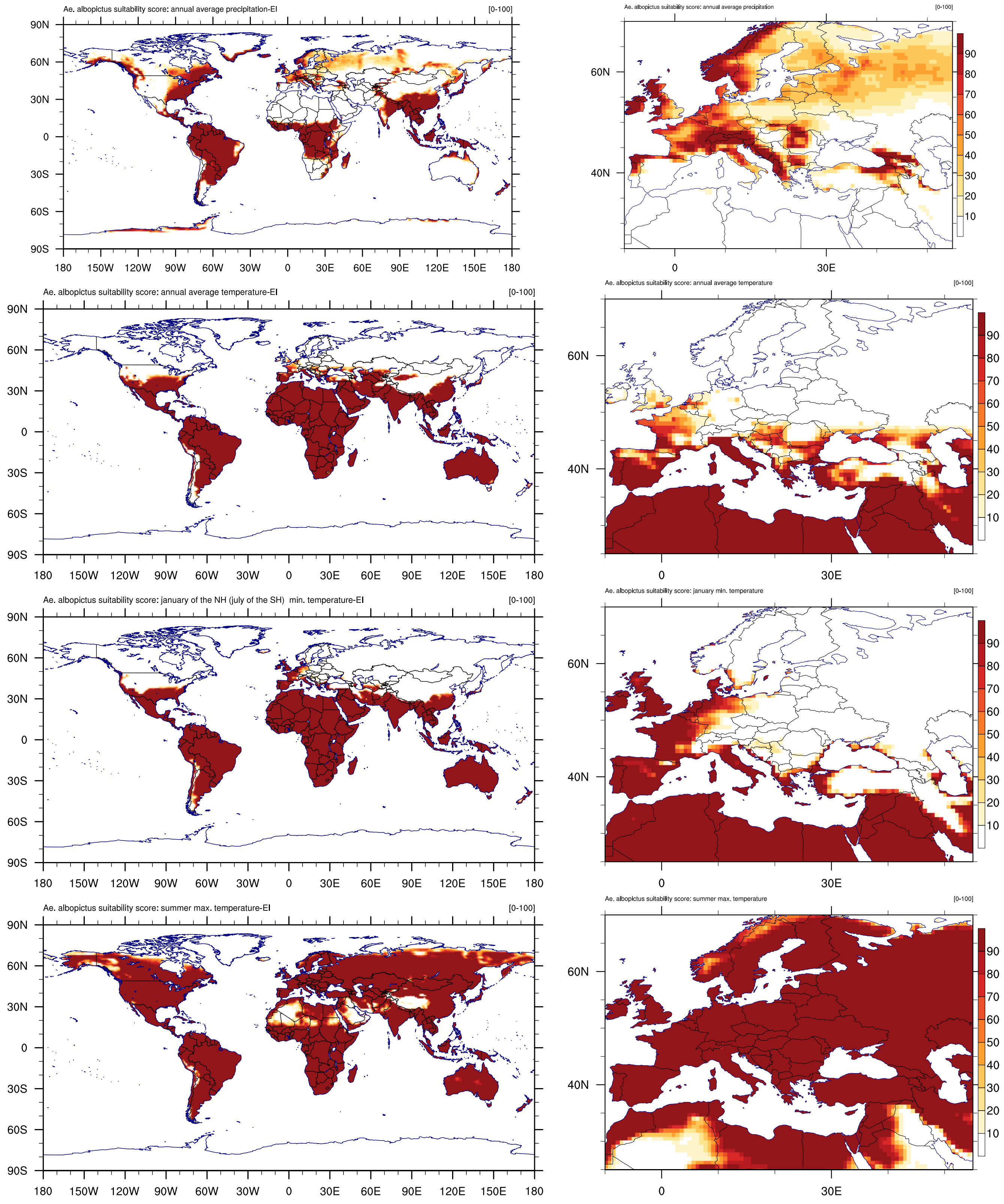

Figure S17: Global and European suitability scores for each of the factors used in the vector distribution model for the period 2000 to 2009, based on ERA-Interim data.

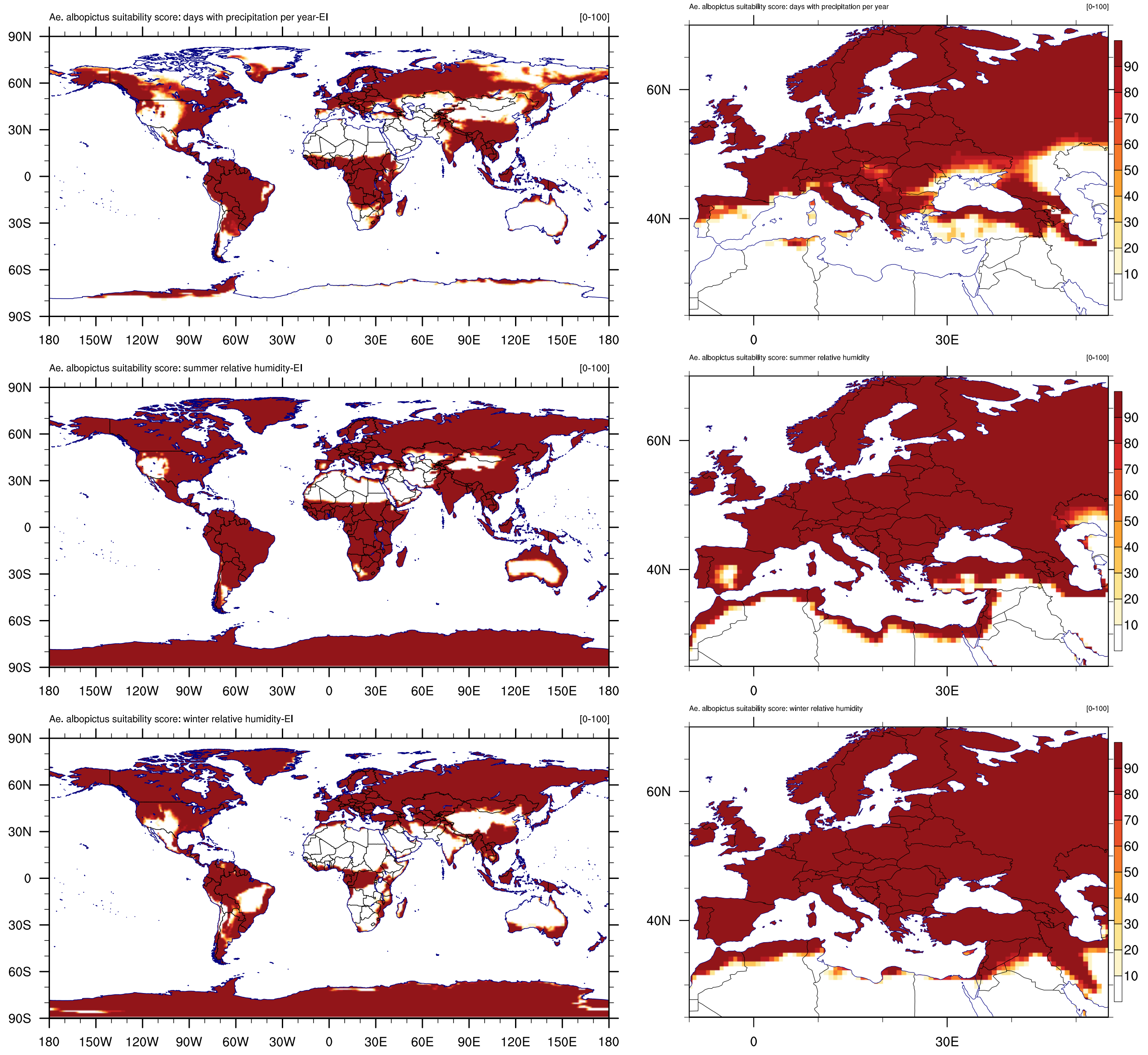

Figure S18: Global and European suitability scores for each of the factors used in the vector distribution model for the period 2000 to 2009, based on ERA-Interim data.

6.1 Differences in habitat suitability between EMAC and ERA-Interim

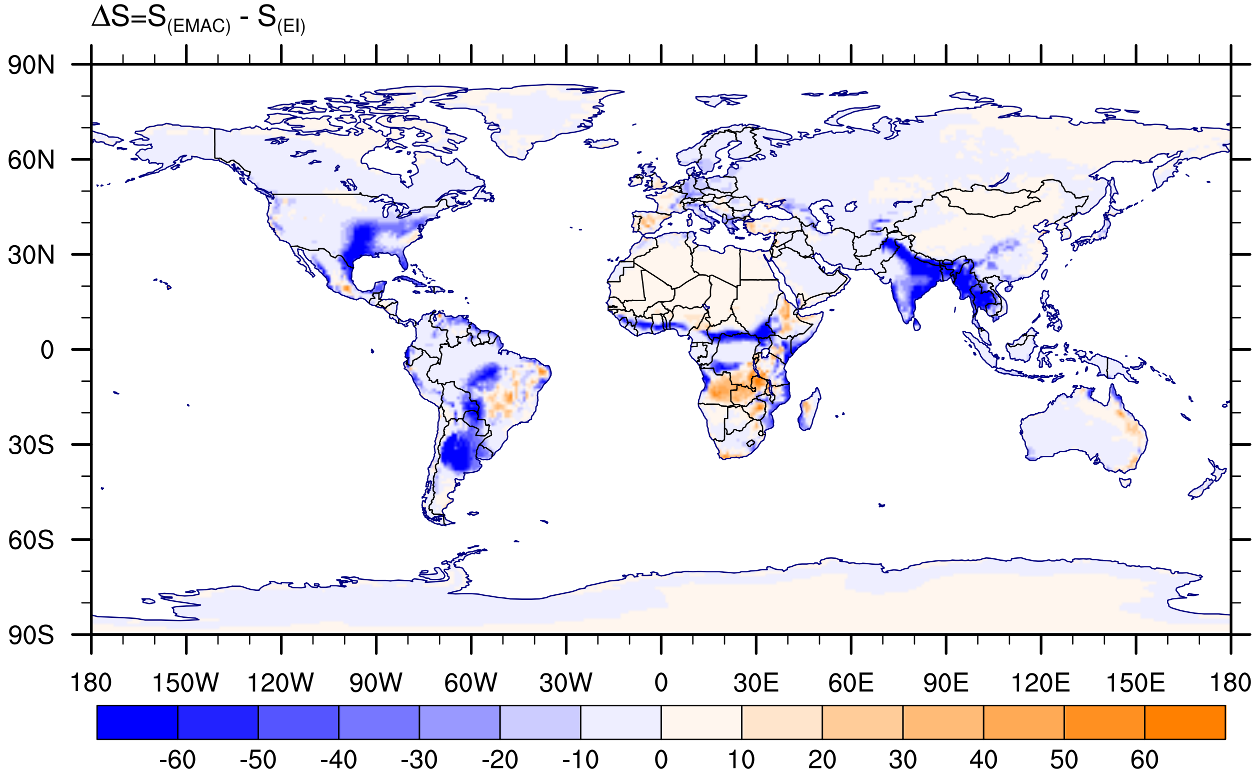

Figure S19: Habitat suitability: Difference between the (re-gridded) EMAC results and the ERA-Interim data for the recent past (2000-2009).

7 Fewer constraints: The three-variable vector distribution model

In the manuscript the habitat suitability maps were generated using a seven-variable (fuzzy-logic mcda-based) vector distribution model. Here we have included a version using three meteorological variables (precipitation, January of the NH (July of the SH) minimum temperature and summer maximum temperature), identical to recently published studies [1, 2], with the exception that the predictor variables are combined via a simple geometric rather than an arithmetic mean combination. The latter was chosen so that we can have a fair comparison against the the seven-variable vector distribution model utilized in the manuscript, which estimates the habitat suitability using a geometric mean combination. Below are presented results showing the relevant results (over European continent) for both the recent past (2000-2009) and the mid-century projections (2045-2054). The corresponding absolute differences in habitat suitability are also presented below.

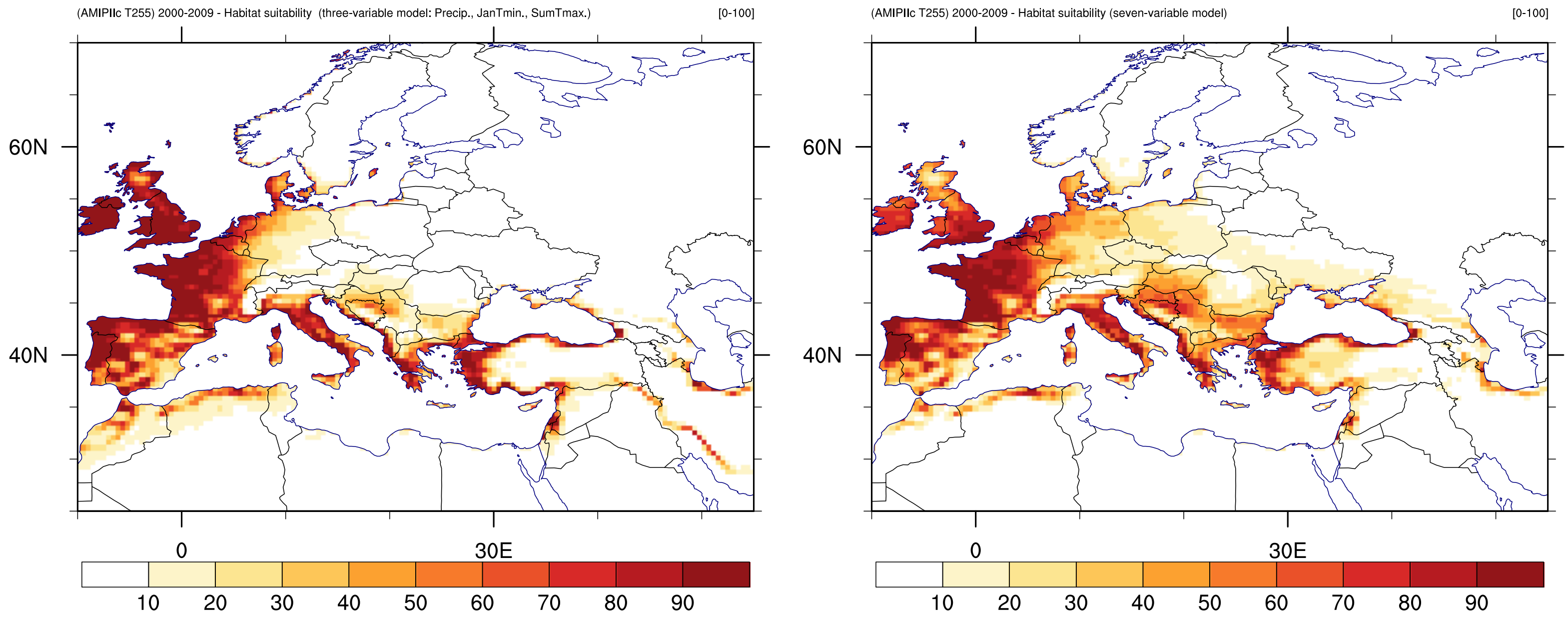

Figure S20: The figure on the left shows the recent past habitat suitability, focused in Europe, estimated using three meteorological variables (precipitation, January of the NH (July of the SH) minimum temperature and summer maximum temperature, as in [1, 2]). On the right the result using the seven-variable vector distribution model described in the manuscript. Despite several differences, there is a strong spatial (grid-space) correlation between the two results, with a Pearsons r-test correlation coefficient, $r = 0.92\,[99.9\%\,CI,\ p < 0.001]$.

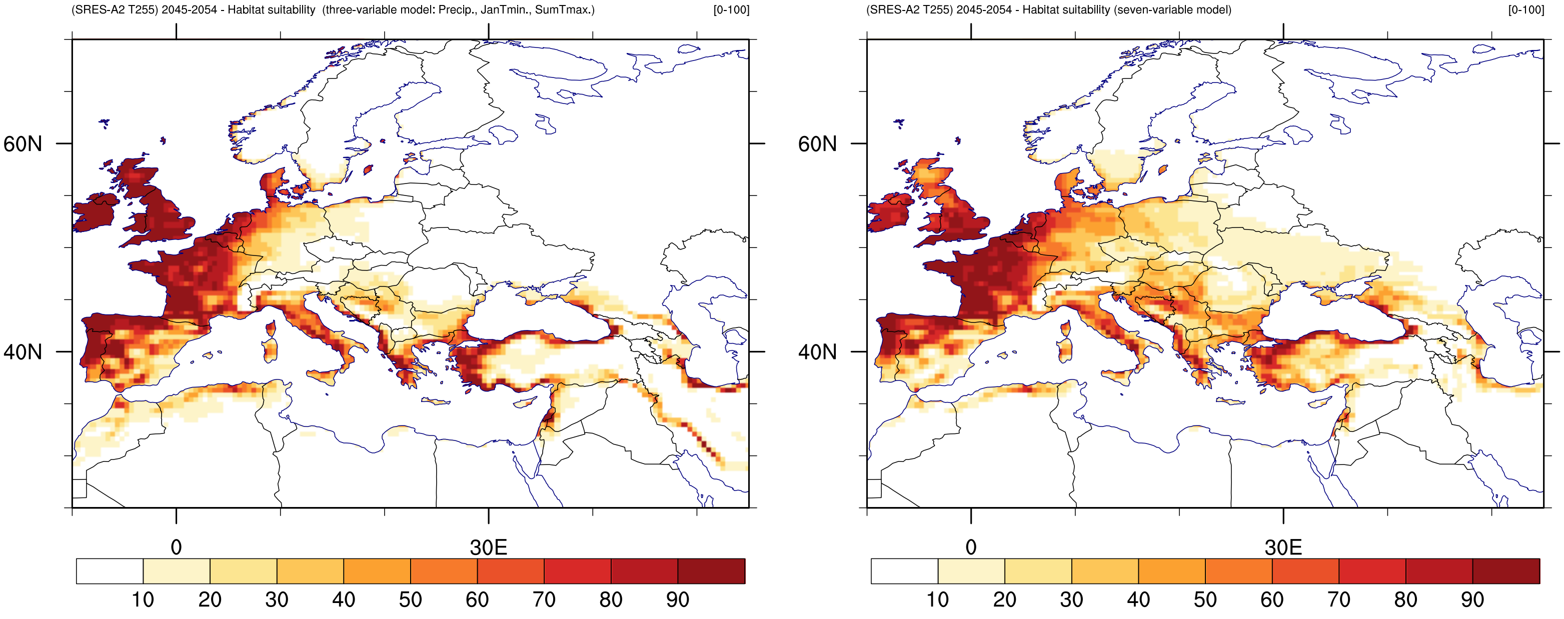

Figure S21: The figure on the left shows the mid-century projections of the habitat suitability, focused in Europe, estimated using three meteorological variables (precipitation, January of the NH (July of the SH) minimum temperature and summer maximum temperature, as in [1, 2]). On the right the result using the seven-variable vector distribution model described in the manuscript. Despite several differences, there is a strong spatial (grid-space) correlation between the two results, with a Pearsons r-test correlation coefficient, $r = 0.93\,[99.9\%\,CI,\ p < 0.001]$.

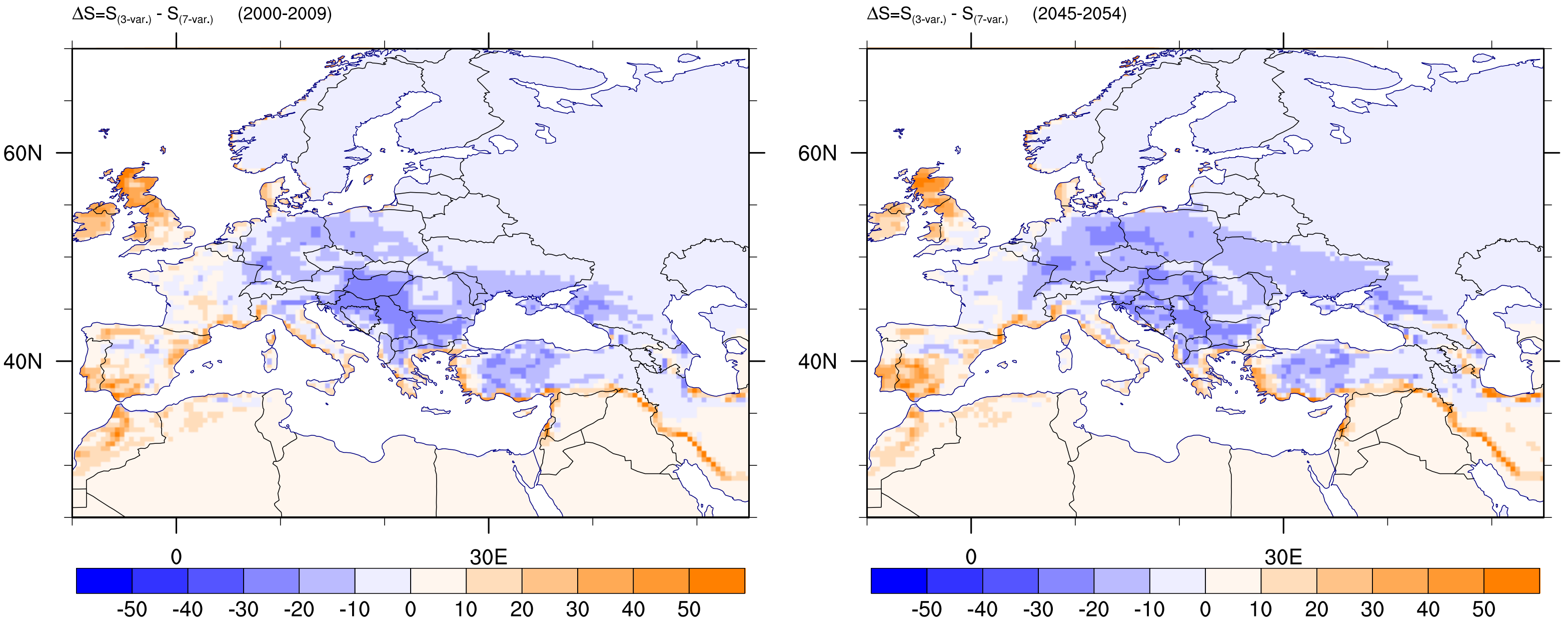

Figure S22: Absolute differences between the three- and seven-variable vector distribution models for both simulation periods.

Although the habitat suitability patterns from the two vector distribution models seem to be very similar (same colour scale is used), the two maps in Figure S22 indicate that the three-variable model estimates higher habitat suitability values in the northern British Isles, south-east Iberian peninsula, southern France, Greece and western Turkey, while the seven-variable model has a tendency to predict higher values in Central and southeastern Europe.

8 CMIP5: Area weighted mean near surface temperature

The graphs below show the area weighted mean (near surface) temperature for recent and future periods, respectively. We have not excluded areas with hsi less than 10% in this case, in contrast to the result presented in the manuscript.

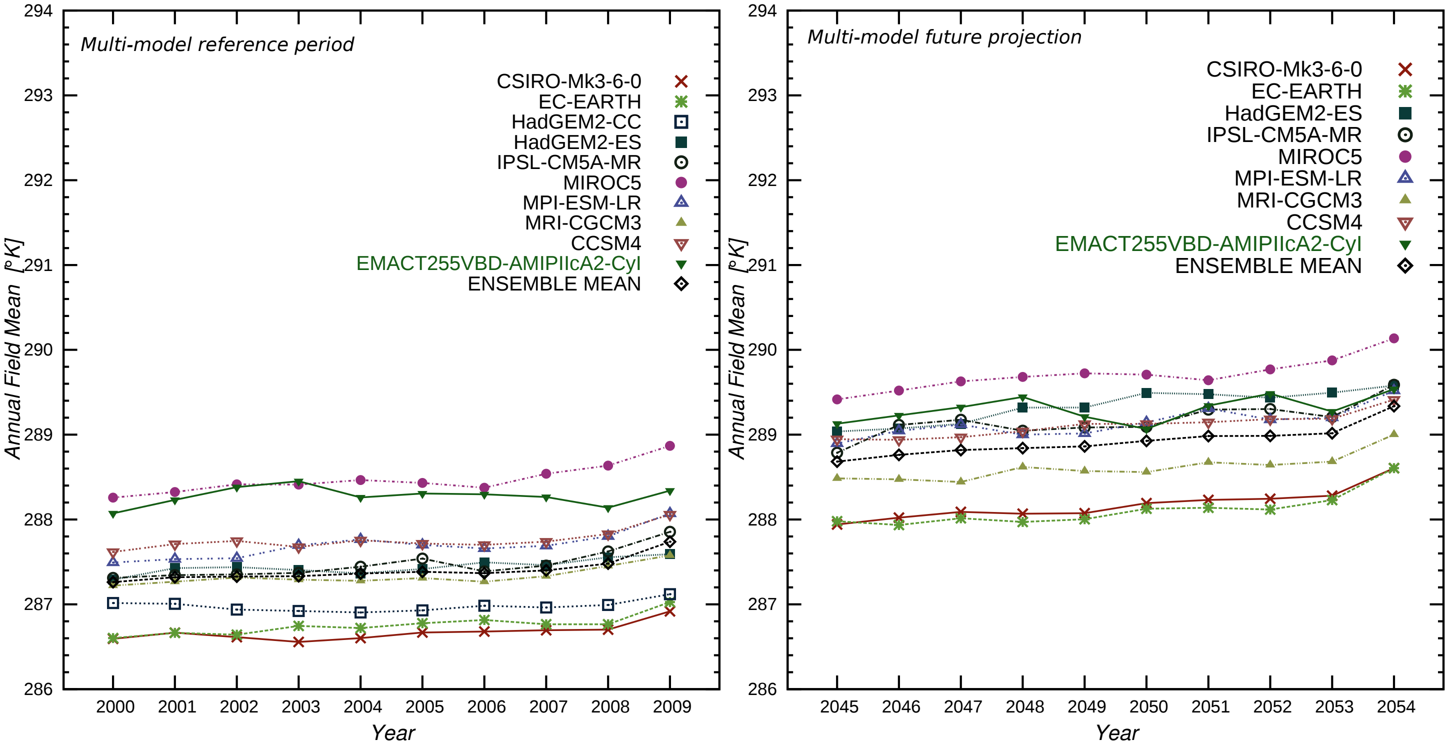

Figure S23: Left: Area weighted mean (near surface) temperature for the recent period 2000-2009 for the CMIP5 ensemble along with the bi-linearly interpolated EMAC result. Right: Results for the same variable but over the future period 2045-2054.

References

[1] European Centre for Disease Prevention and Control. Development of Aedes albopictus risk maps. Technical report, European Centre for Disease Prevention and Control, 2009. [Accessed 28 Feb., 2014].

[2] Cyril Caminade, Jolyon M. Medlock, Els Ducheyne, K. Marie McIntyre, Steve Leach, Matthew Baylis, and Andrew P. Morse. Suitability of European climate for the Asian tiger mosquito Aedes albopictus: recent trends and future scenarios. *Journal of The Royal Society Interface*, 9(75):2708–2717, 2012.
